# Linking Chemical Structure and Coherence Times of Molecular Spin Qubits

**DOI:** 10.1021/jacsau.6c00489

**Published:** 2026-06-18

**Authors:** Sarah Suchaneck, Lorenzo Tesi, Joris van Slageren, Andreas Köhn

**Affiliations:** † Institute for Theoretical Chemistry, 9149 University of Stuttgart, Pfaffenwaldring 55, Stuttgart D-70569, Germany; ‡ Institute of Physical Chemistry, University of Stuttgart, Pfaffenwaldring 55, Stuttgart D-70569, Germany; ∥ Center for Integrated Quantum Science and Technology (IQST), University of Stuttgart, Pfaffenwaldring 55, Stuttgart D-70569, Germany

**Keywords:** molecular spin qubit, quantum coherence time, nuclear spin diffusion, spin diffusion barrier, spin echo, electron paramagnetic resonance, cluster-correlation expansion

## Abstract

The electron spin is a natural qubit platform whose properties, when embedded in a molecule, can be tuned by synthetic chemistry. However, the quantum properties can be exploited only over a limited time scale, and lengthening the coherence time remains a significant challenge. Nuclear spin diffusion is one of the principal sources of decoherence, but a complete removal of spin-bearing nuclei is hardly feasible in practice, and therefore, strategies to mitigate their impact are essential. Despite considerable efforts to screen the chemical space for improved molecular spin qubits (MSQs) as well as advances in simulation techniques to understand the physical details of the decoherence processes, a broader picture bringing together the chemical and physical views of this phenomenon is still lacking. In this work, we fill this gap by analyzing the decoherence induced by a nuclear spin bath of protons through a parameter space screening approach based on the analytic pair product approximation. As specific cases, we discuss [Cu­(dbm)_2_] (H-dbm = dibenzoylmethane) and (PPh_4_)_2_[Cu­(mnt)_2_] (mnt^2–^ = maleonitriledithiolate) diluted into a nonmagnetic crystalline matrix. The analysis reveals the geometric and statistical conditions that lead to strong contributions to decoherence and enables us to identify the nuclear pairs that are most relevant to decoherence effects. This allows formulating new design rules for proton-containing MSQs and systematically reveals potential avenues for their improvement. Most importantly, our analysis emphasizes that long coherence times require a proper design of the environment in which the MSQ is embedded, while nuclear spins on the MSQ itself are less important.

## Graphical abstract

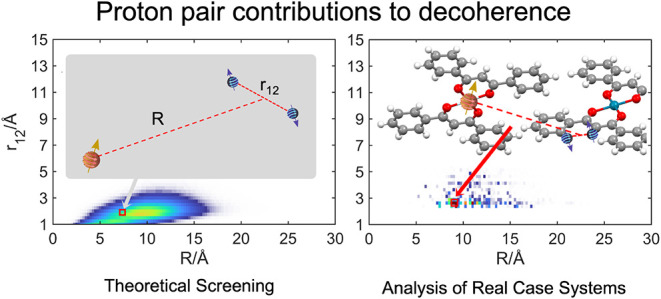

## 1 Introduction

Molecular spin qubits (MSQs) are promising candidates as the core units for future quantum technologies.^1^ They utilize the electronic spin to encode the qubit states and are an interesting alternative to electron spin defects in solid-state systems, such as nitrogen vacancy centers or phosphorus atoms in silicon, because they can be chemically tuned with high flexibility.^2^ This flexibility also enables their deposition on surfaces^3^ and integration into a wide range of host environments.^4^ The practical utility of a qubit depends on its quantum coherence time, defined by the time scale over which it retains its coherent quantum state: achieving long coherence times is essential for performing quantum operations reliably.^1^ For this reason, significant experimental efforts have been directed toward understanding the causes of decoherence by addressing specific parameters such as the center carrying the spin,^5^ the molecular geometry,^6^ the size of the counterion,^7^ and the host matrix.^8,9^ As a result, spin–lattice relaxation, electron–electron dipolar coupling, and hyperfine interactions have been identified as the main sources of decoherence.^10^

Manipulation of the electron spin perturbs the spin population away from thermal equilibrium. The characteristic time scale over which the equilibrium population is restored through energy exchange with the lattice is known as spin–lattice relaxation time (*T*_1_). This relaxation process originates from the coupling between the spin system and lattice vibrations, which induce fluctuations of the local crystal field, spin–orbit coupling, or g-tensor anisotropy.^11^ As a consequence, energy and phase information associated with the qubit state are irreversibly dissipated into the phonon bath. The efficiency of this process depends on the spin–phonon coupling, which is determined by factors such as the phonon density of states at the relevant energies, the spin–orbit coupling, the rigidity of the molecular framework, and the magnetic anisotropy of the system.^12,13^ Longer spin–lattice relaxation times can therefore be achieved by using light elements^14^ and rigid lattices,^15,16^ by controlling the degree of s-mixing into spin-bearing orbitals,^17–20^ or by exploiting specific symmetries.^21–24^ A detailed understanding of the mechanisms that drive spin–lattice relaxation has recently been achieved through the development of first-principle models capable of predicting its behavior,^25,26^ complemented by experimental investigations of both acoustic and optical phonons in MSQ crystals.^27,28^ In most cases, however, spin–lattice relaxation limits the coherence times only when approaching room temperature.^6,29^

Electron–electron dipolar coupling induces decoherence due to fluctuating nearby electron spins. Decoherence may occur either through direct flip-flop processes involving the qubit spin itself or through flip-flops and relaxation processes within the surrounding electron spin bath, which dynamically modulate the local magnetic field experienced by the qubit and induce spectral diffusion.^30^ Electron–electron dipolar coupling can be effectively mitigated by increasing the distance between the electron spins, which can be achieved by diluting the MSQs in a nonmagnetic matrix, either by cocrystallization with a diamagnetic molecular analogue or by dilution in a solvent or glass matrix.^31,32^

Hyperfine-induced decoherence originates from magnetic coupling between the electron spin and the surrounding nuclear spin bath. Similar to the electron spin bath, the nuclear spin bath generates a dynamic local magnetic field that modulates the qubit phase through spectral diffusion. Although the effect of individual nuclei on decoherence is generally weaker due to the smaller nuclear magnetic moments and slower spin dynamics compared to electron spins, the high abundance of magnetic nuclei in molecular systems often makes nuclear spin flip-flops the dominant limitation to coherence in diluted systems below 100 K. Empirically, nuclei with larger gyromagnetic ratios lead to faster coherence decay, and ^1^H has been identified as one of the main contributors to electron spin dephasing, also because of its high abundance in molecular systems.^33^ Strategies to mitigate decoherence, such as exploiting clock transitions formed by zero-field or hyperfine splitting,^34,35^ have so far shown to increase coherence times only to a limited extent. Instead, efforts to completely remove proton spins have yielded record coherence times for organic radicals^14,36^ and transition metal-based MSQs.^29,37–39^ However, although at first glance this strategy appears straightforward, complete removal is often impractical, if not impossible, in realistic experimental scenarios, because of the ubiquitous presence of protons in molecules and their environment.

Therefore, understanding how coherence in MSQs can coexist with proton spins becomes imperative. Hence, the aim of this work is to identify the link between the chemical structures of MSQs and the physical mechanisms according to which nuclear spins induce loss of coherence in electron spins. We seek to understand and extend design criteria for improved coherence times, thereby revisiting and rationalizing the experience from previous experimental work^29,37,40–44^ and theoretical studies.^8,32,45–54^ While previous theoretical studies have focused on the role of the matrix^8,45^ and spin dilution,^32^ or purely intramolecular effects,^46,55^ this work aims to analyze the role of proton spins in both the MSQ and its environment with particular focus on the geometric conditions that diminish or enhance coherence times. To achieve this aim, we employed a rather simple yet accurate analytic model for the decoherence process as initially put forward by Witzel et al. in the context of spin defects in solid-state materials.^56,57^ Following Jeschke,^50^ we adopted the name analytic pair product approximation (APPA) for this approach. It can be understood as a second-order cluster-correlation expansion^58–61^ of the electron spin echo envelope modulation (Hahn echo signal) in the high-field limit. In this limit, which is reached for magnetic fields above ∼1 T (echo detection at Q-band),^51^ contributions of single-nuclear spins due to the anisotropy of the hyperfine interaction are quenched^57^ (these single-nuclear spin flip processes only modulate the Hahn echo signal, but do not contribute to its decay^50^). The expansion then only contains contributions from flip-flop processes of pairs of bath spins, and an analytic expression for these contributions can be derived.^50,56,57^ In the context of molecular systems, this method was already successfully applied by Lenz et al. to Cu­(II)-based MSQs, demonstrating very good agreement with the experimental spin relaxation decay observed by pulsed electron paramagnetic resonance (EPR) spectroscopy.^51^ Jeschke et al. employed this method to study the refocusing effectiveness of Carr–Purcell dynamic decoupling pulse sequences on nitroxyl radical derivatives in solution^50^ and to study the coherence times of trityl radical derivatives in solution under microwave irradiation.^52^ The accuracy of the second-order expansion, albeit without resorting to the analytic high-field limit, is also confirmed by the work of Stoll and co-workers, who accurately reproduced the decay curves for several organic radical molecules.^47^ The approach also allowed studying the impact of protons on the coherence times of a nitroxyl radical in a frozen solid glassy matrix for different levels of deuteration^48^ and only requires higher-order terms for the more intricate case of methyl groups, where tunnelling introduces significant higher-order correlations.^49,52^

Here, we analyze the electron spin decoherence induced by the nuclear spin bath consisting of protons with $I=\frac{1}{2}$ . We consider the Hahn echo experiment and, as a step beyond previous works,^47–52^ exploit the straightforward structure of the APPA equations to systematically analyze the proton pair contributions to decoherence as a function of their geometric parameters. This parameter space screening approach enables us to understand how the decay of the Hahn echo signal emerges from the various pair contributions and to identify the specific nuclear spin pairs that contribute the most to decoherence. The universality and predictive power of these results are then evaluated by comparing them to the specific case of [Cu­(dbm)_2_] (dbm = dibenzoylmethane) and (PPh_4_)_2_[Cu­(mnt)_2_] (mnt = maleonitriledithiolate) doped into their respective isostructural diamagnetic host crystal, for which very accurate measurements are available.^29,51^ We finally test the predicted impact of partial substitutions of protons on the coherence times.

## 2 Theory

In the Hahn echo experiment,^30^ a sample is placed inside a homogeneous magnetic field, which defines a quantisation axis for the spin states. At time zero, a π/2 excitation pulse is applied to the sample, creating a coherent superposition state of the electron spins, after which their states evolve freely for a waiting time τ. They are then refocused with a π pulse and an echo signal is detected after another time period τ. The intensity of the Hahn echo signal *S*(2τ) is the central quantity detected in this experiment. Its value decays with increasing τ due to processes affecting the electron spin state, which in a magnetically diluted sample at low temperature are solely flip-flop processes of nuclear spin pairs weakly interacting with the electron spin.^32^ Additional decoherence at low temperatures can be induced by tunnelling processes, for instance tunnelling of methyl groups,^49,52^ which are not considered in the present work. The APPA models the Hahn echo decay by considering the product of these flip-flop contributions from all possible pairs of nuclei in the spin bath:^50,56,57^

1 $$S\left(2\tau \right)=\prod_{k>l}s_{\mathrm{kl}}^{\left(2\right)}\left(2\tau \right)$$

Restricting the discussion to the case of spin- $\frac{1}{2}$ nuclei, the contributions from each pair of bath nuclei are

2 $$s_{\mathrm{kl}}^{\left(2\right)}\left(2\tau \right)=1-\Lambda_{\mathrm{kl}}\;\sin^{4}\left(\frac{\left(2\tau \right)\omega_{\mathrm{kl}}}{4}\right)$$

and consist of the modulation depth

3 $$\Lambda_{\mathrm{kl}}=4\frac{c_{\mathrm{kl}}^{2}}{{\left(1+c_{\mathrm{kl}}^{2}\right)}^{2}},\;\mathrm{with}\;c_{\mathrm{kl}}=\frac{A_{k}-A_{l}}{b_{\mathrm{kl}}}$$

and the corresponding modulation frequency (or nuclear zero-quantum frequency^50^) of the flip-flop process:

4 $$\omega_{\mathrm{kl}}=\frac{1}{2}\sqrt{b_{\mathrm{kl}}^{2}+{\left(A_{k}-A_{l}\right)}^{2}}=\frac{1}{2}\left|b_{\mathrm{kl}}\right|\sqrt{1+c_{\mathrm{kl}}^{2}}$$

Both quantities Λ_*kl*_ and ω_*kl*_ depend on the mutual interactions between the spins, which are assumed to be purely dipolar. These are the (dipolar) hyperfine interactions between the central spin and the two nuclei under consideration, *A*_*k*_ and *A*_*l*_, and the dipolar interaction of the two nuclei, *b*_*kl*_:

5 $$A_{k}=\gamma_{S}\gamma_{n}\hbar \frac{1-3\;\cos^{2}\left(\theta_{k}\right)}{r_{k}^{3}},,b_{\mathrm{kl}}=-\gamma_{n}^{2}\hbar \frac{1-3\;\cos^{2}\left(\theta_{\mathrm{kl}}\right)}{r_{\mathrm{kl}}^{3}}$$

Here, *r*_*k*_ is the distance between the spin center and nucleus *k* and *r*_*kl*_ the distance between nuclei *k* and *l*, respectively; *θ*_*k*_ and *θ*_*kl*_ are the angles of the corresponding distance vectors with the magnetic-field direction, assuming an alignment of all spins along the applied magnetic field (high-field approximation). The prefactors consist of the gyromagnetic ratio of the proton γ_n_ and the gyromagnetic ratio of the spin center $\gamma_{S}=\frac{\mu_{B}}{\hbar }g_{\mathrm{eff}}$ , where μ_B_ is the Bohr magneton, ℏ the reduced Planck constant, and *g*_eff_ the projection of the g tensor along the direction of the magnetic field (see Supporting Information Section S1 for details). It should be noted that the field strength does not enter any of the above-described expressions. Indeed, the decoherence introduced by flip-flop processes of nuclear pairs is magnetic-field independent.

The modulation depth Λ_*kl*_ is the amplitude of the Hahn echo signal modulation contributed by a single-nuclear pair. Its magnitude is set by the ratio *c*_*kl*_ = (*A*_*k*_–*A*_*l*_)/*b*_*kl*_ (see eq 3), which relates the energy splitting of the αβ and βα states of the nuclear spin pair to their off-diagonal coupling.^50^ Λ_*kl*_ tends to zero, either in the case |*A*_*k*_–*A*_*l*_| ≫ *b*_*kl*_, i.e., when the hyperfine couplings of the two nuclei are very different, which effectively quenches the flip-flop process, or in the case |*A*_*k*_–*A*_*l*_| ≪ *b*_*kl*_, which is reached when the hyperfine couplings are very similar such that the flip-flop process does not induce any changes of the effective field for the electron spin. In addition, a strong coupling *b*_*kl*_ ≫ |*A*_*k*_–*A*_*l*_| will quench the flip-flop process by giving preference to fixed superpositions of the αβ and βα states of the proton pair. The modulation depth reaches a maximum of Λ_*kl*_ = 1 for |*c*_*kl*_| = 1, when the absolute value of the hyperfine coupling difference equals the dipolar interaction strength between the nuclear spins, i.e., they fulfill the ‘matching condition’^50^ |*A*_*k*_–*A*_*l*_| = |*b*_*kl*_|.

The modulation frequency ω_*kl*_ can be interpreted as the oscillation frequency of the flip-flop process and sets the time scale over which the nuclear spin pair contributes to the Hahn echo signal. It also depends on the dipolar coupling between two nuclear spins and the difference in their hyperfine couplings to the electron spin (eq 4). Specifically, it increases when the dipolar coupling or the hyperfine coupling differences are large. Close to the matching condition, when *c*_*kl*_^2^ ≈ 1, its value is mainly determined by the nuclear dipole coupling, as evident from the second equality in eq 4.

The individual pair contributions (eq 2) are periodic, and the specific sin^4^([2τ]­ω_*kl*_/4) behavior is rooted in the Hahn echo pulse sequence, where the π pulse inverts slow and fast dephasing components and cancels faster oscillating contributions.^57^ Jeschke^50^ has also pointed out an interesting relation of this expression to filter functions.^62^ The product of the pair contributions becomes nonperiodic when all of the pairs from the spin bath are considered, because the individual contributions possess different frequencies. Obviously, each pair contribution depends on the strengths of the dipolar interactions between the three spins, which, in turn, are governed by their magnetic anisotropy and spatial arrangement. This includes both the distances between the spins as well as their alignment with respect to the applied magnetic field and emphasizes the role of both molecular structure and magnetic-field alignment in the decoherence process.

## 3 Parameter Space Screening

To systematically investigate the impact of the spatial arrangement of a proton pair relative to the central spin and analyze the individual pair contributions (eq 2), we use the following coordinates: The three spin centers define a plane into which we place the *x* and *y* axes of a coordinate system, as shown in Figure 1a. The arrangement of the spins is then described by Jacobi coordinates, with *R* denoting the distance of the electron spin center to the midpoint of the nuclear spin pair, while *r*_12_ is the distance between the two nuclear spins, see Figure 1a. The angle between the two distance vectors is called α. The orientation of the plane spanned by the three nuclei with respect to the applied magnetic field **B**_0_ can be described by the polar angles θ and ϕ, as indicated in Figure 1b. Note that a third Euler angle is not required as a rotation of the system around the magnetic-field axis does not change any interaction. For further details, see Supporting Information Sections S2.1 and S2.2.

**Figure 1:**
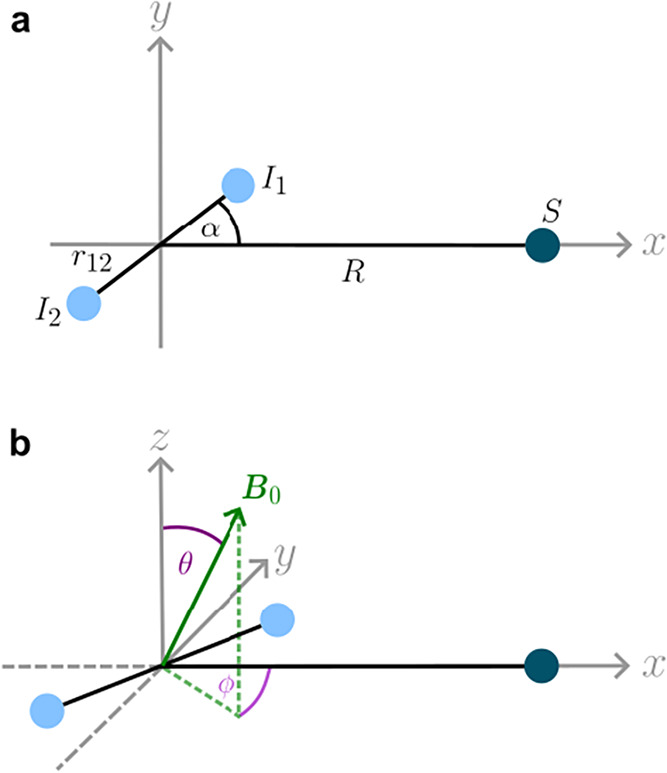
Representation of the coordinate system used to analyze the individual contributions to the Hahn echo decay. The three-particle spin system consists of a central electron spin *S* and two nuclear spins *I*_1_ and *I*_2_. (a) Interspin distances are described by Jacobi coordinates *R*, *r*_12_, and α. (b) Orientation with respect to the external magnetic field **B**_0_ is described by polar coordinates.

According to this scheme, each pair contribution (eq 2) only depends on five spatial parameters, *R*, *r*_12_, α, θ, and ϕ. In the following, we will analyze the modulation depth Λ_12_ and the modulation frequency ω_12_, as well as the resulting echo contribution *s*_12_^(2)^(2τ), as functions of these parameters, in order to identify the geometric conditions leading to decoherence. From eqs 3 and 5, we deduce that the most important parameters are the distances *R* and *r*_12_, which mainly determine the strength of the dipolar interactions. Particularly, the modulation depth becomes small if there is a large misfit in the order of magnitude of these interactions. We have to bear in mind, however, that it is the difference in the hyperfine interactions rather than their absolute values that enters into the equations. This leads to a subtle dependence of Λ_12_ and ω_12_ on the angle α. For instance, for a T-shaped arrangement (α = 90° or 270°) and a perpendicular orientation of **B**_0_ (θ = 0, ϕ arbitrary), the hyperfine interactions are identical, and the modulation depth Λ_12_ becomes zero. This highly symmetric arrangement will therefore not contribute to the decoherence. Tiny distortions of this arrangement, however, either by changing α or the orientation relative to **B**_0_, may be sufficient to reach the matching condition |*A*_1_–*A*_2_| = |*b*_12_|, which results in a large value for Λ_12_ and a possibly strong pair contribution to the decoherence process. On the other hand, in regions of the parameter space where the magnitudes of the hyperfine couplings and the nuclear spin–spin interaction differ substantially, the matching condition is met only for a tiny range of α values (or orientations of the magnetic field), as shown in the Supporting Information Sections S2.3 and S2.4, and we consider them of little importance for the further discussion. These preliminary considerations already suggest that a sound discussion of the geometric conditions leading to significant pair contributions to the decoherence can be based on the parameters *R* and *r*_12_, while averaging over all other parameters α, θ, and ϕ numerically (as described in the Supporting Information Section S2.5).

The resulting averaged quantities are shown in Figure 2a,b as functions of *R* and *r*_12_. Note that configurations with values of *r*_1_ or *r*_2_ smaller than 1 Å have been omitted in the averaging over α to exclude contributions from unphysically close-lying spin centers. As demonstrated in Supporting Information Sections S2.3 and S2.4, the main features of the plots in Figure 2 are very similar for all α values and magnetic-field orientations, with only a few exceptional configurations that do not significantly contribute to the average (for instance, the dipolar interactions, eq 5, vanish at the ‘magic angle’ relative to the applied magnetic field **B**_0_).

**Figure 2:**
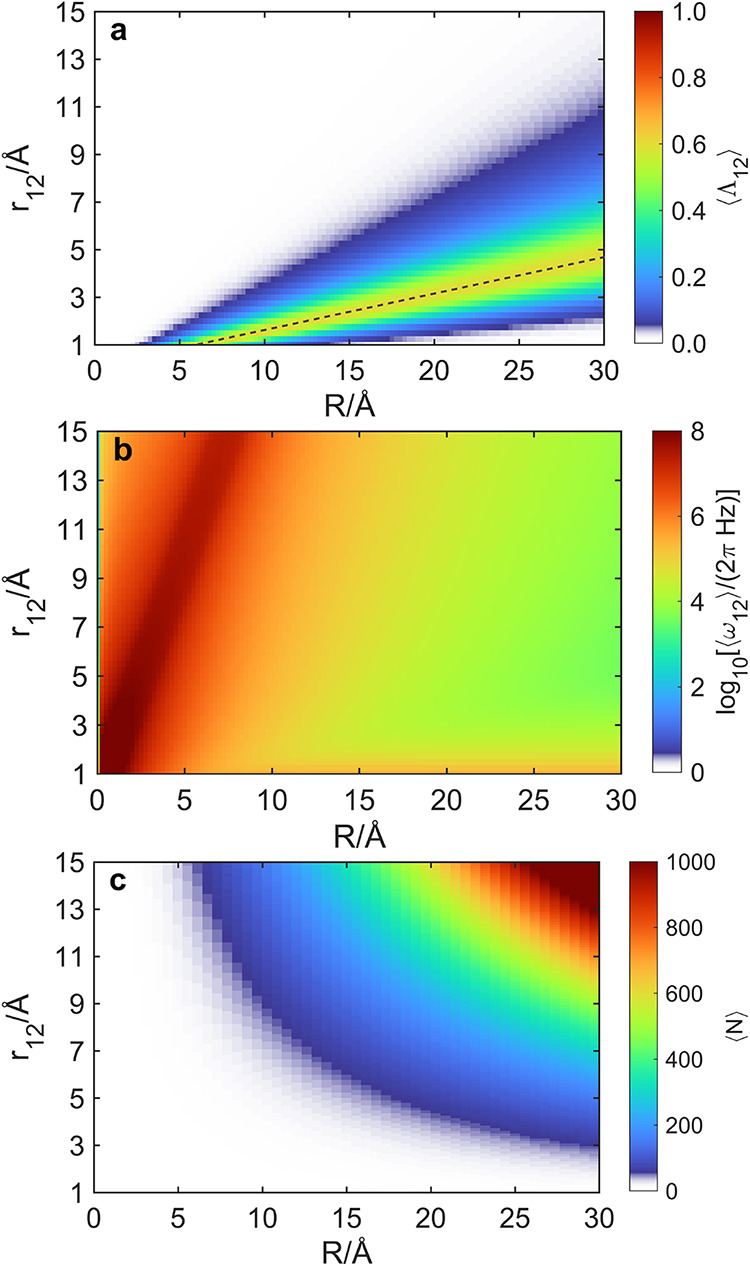
Nuclear spin pair contributions to the Hahn echo decay as a function of the electron–nuclear distance *R* and the internuclear distance *r*_12_ (averaged over the Jacobi angle α and all orientations relative to the magnetic field). (a) Modulation depth Λ_12_ (the dotted line indicates the maximum and refers to the relation given in eq 6) and (b) modulation frequency ω_12_. Panel (c) shows the average number of protons available for a given (*R*, *r*_12_) configuration, normalized to a bin size of 0.25 × 0.25 Å.

The modulation depth Λ_12_ assumes significant values (Λ_12_ > 0.05) in a fan-like area that broadens for larger values of *R*. It shows a maximum along a line given by the relation

6 $$\frac{r_{12}}{R}=C{\left(\frac{\gamma_{n}}{\gamma_{S}}\right)}^{1/4}$$

where the additional constant *C* weakly depends on the other parameters (α, θ, ϕ), as discussed in the Supporting Information Section S2.6. The origin of this area of large modulation strength is the matching condition |*A*_1_–*A*_2_| = |*b*_12_|. Notably, for *R* < 5 Å, the matching condition is only met by unphysically short proton pair distances *r*_12_ < 1 Å, effectively quenching any influence on the decoherence process of protons that are closer to the electron spin center than ∼4 Å. This phenomenon is known as the nuclear spin diffusion barrier,^38,47,63^ a notion commonly applied to dynamic nuclear polarization (DNP) experiments, where the electron spin polarization has to be transferred efficiently to the nearby nuclear spins; the process is closely related to the coherence decay of MSQs and has therefore also been used in this context.^38,47,63^

The values of ω_12_, which set the time scale at which the nuclear spin pair contributes to the decoherence process, span a wide range, see Figure 2b. For short distances *R* < 5 Å, we find very high values in the 10 MHz range, and there is a maximum along *r*_12_/*R* = 2. For this ratio of distances and α approaching 0 or 180°, the distance between the spin center and one of the nuclei becomes very small, leading to a large hyperfine contribution *A*_*k*_ and thus a large ω_12_. All of these configurations with large ω_12_, however, are of little relevance for the Hahn echo decay, as the modulation depth is very small in these cases. For configurations that give rise to high modulation depth (the fan-like region shown in Figure 2a), the value of ω_12_ is mainly determined by the dipolar coupling of the nuclear spins, as in this limit, ω_12_ ≈ |*b*_12_|/√2. It rapidly decays for larger *R* and *r*_12_, reaching the 1 kHz regime around *R* = 10 to 15 Å and *r*_12_ ≈ 3 Å. From this, we can conclude that nuclei farther away from the electron spin center become only relevant at long time scales. A similar observation was also reported by Jeschke.^50^

A further important variable is the number of available nuclear pairs within the range given by [*R*, *R* + Δ*R*] and [*r*_12_, *r*_12_ + Δ*r*_12_]. We can estimate this value, assuming a constant density of protons ρ_n_, by the expression:

7 $$N\left(R,r_{12}\right)=\frac{1}{2}\rho_{n}^{2}{\left(4\pi \right)}^{2}R^{2}r_{12}^{2}\Delta R\Delta r_{12}$$

where the factor 1/2 eliminates double-counting. A graphical representation of this function is shown in Figure 2c, using a proton density of 0.037 Å^–3^, which corresponds to typical molecular systems (see Section 4). The graph shows that there are only a few pairs for each (Δ*R*, Δ*r*_12_) interval in the region up to *R* ∼ 15 Å and *r*_12_ ∼ 5 Å, but their number strongly increases for larger distances.

The actual contribution of the nuclear spin pair to the Hahn echo decay *s*_12_^(2)^(2τ) (eq 2) is a time-dependent function, including both Λ_12_ and ω_12_. As the values of Λ_12_ and ω_12_ are strongly correlated, we cannot simply use their averages to estimate their contribution at a given *R* and *r*_12_. For instance, the high values of ω_12_ seen in Figure 2 originate from a large difference in hyperfine coupling (*A*_1_–*A*_2_), which correlates with small values for Λ_12_. Therefore, we have applied the same averaging procedure to the pair contributions *s*_12_(2τ) as used before for Λ_12_ and ω_12_ (see also Supporting Information Section S2.5) and determined an average for a given configuration (*R*, *r*_12_), denoted ⟨*s*_12_(2τ)⟩ in the following. This quantity is shown in Figure 3 (panels a–c) for different delay times τ. Indeed, within this theoretical model, increasing the interpulse delay time makes it possible to identify which proton pairs contribute to the echo decay at specific time scales.

**Figure 3:**
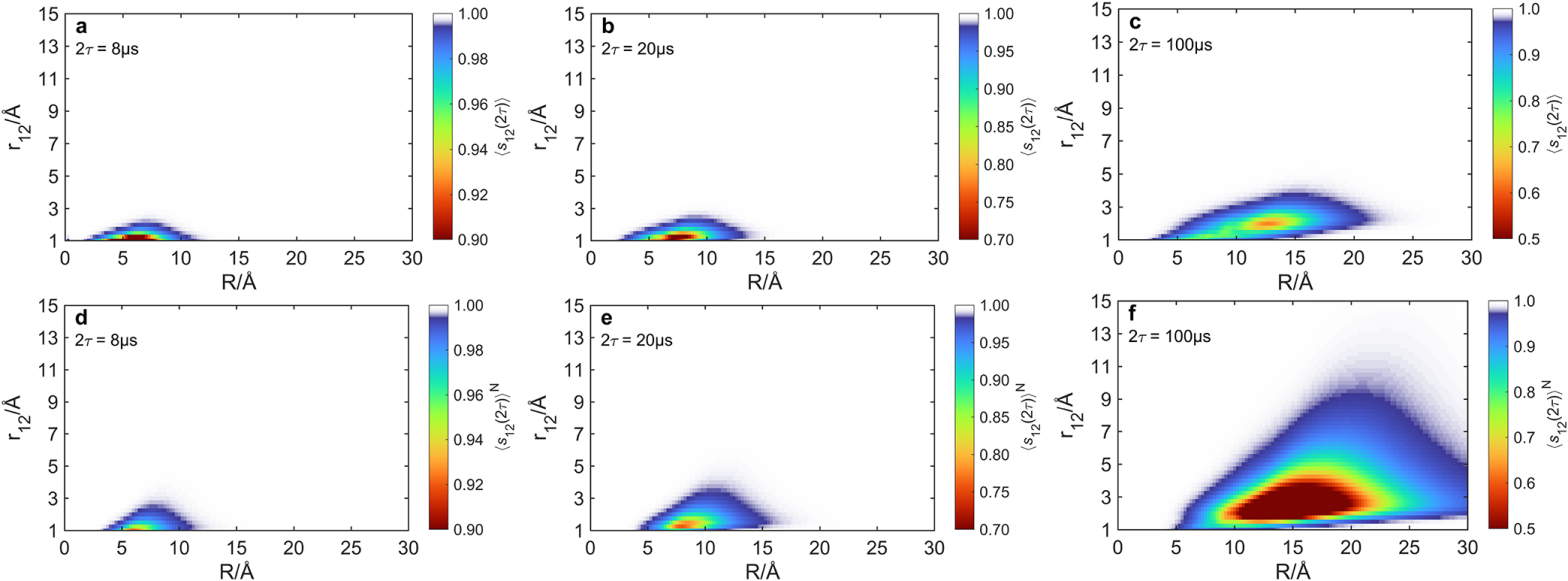
Panels (a–c): Averaged pair contribution to the Hahn echo signal ⟨*s*_12_(2τ)⟩ as a function of the electron–nuclear distance *R* and the internuclear distance *r*_12_ at different delay times. Panels (d–f): Averaged pair contribution raised to the power of average pairs for the given distance ⟨*s*_12_(2τ)⟩^*N*^. Note that the color scale has been adapted for the various delay times to enhance visibility.

For very short delay times, like 2τ = 8 μs (Figure 3a), only proton pairs close to the spin center with *R* up to 12 Å contribute. The matching condition is then met by proton pairs that are in the range of 1 < *r*_12_ < 3 Å. As discussed before, protons that are closer than ∼4 Å to the spin center do not contribute (“diffusion barrier”). With increasing delay time τ, the significant contributions to the coherence decay shift toward larger values of *R* and *r*_12_ (Figure 3b,c), following the region of large modulation depth shown in Figure 2a.

Using the average number of proton pairs, eq 7, we can estimate the overall contribution of pairs at a given distance by ⟨*s*_12_(2τ)⟩^*N*^. Due to this exponential dependence, the impact of the number of pairs (and thus the proton density ρ*_n_*) is significant, in particular for longer delay times when a larger part of the proton bath around the electron spin center begins to contribute. This is shown in Figure 3 (panels d–f).

In the region close to the spin center, the average number of protons is still very low, and consequently, there is no significant effect of the proton number on the signal for short delay times up to 2τ = 8 μs: for both ⟨*s*_12_(2τ)⟩ and ⟨*s*_12_(2τ)⟩^*N*^, significant contributions are in a very similar range, with the latter being shifted to marginally larger *R* and *r*_12_ values (compare Figure 3a,d).

For slightly longer times, for example, 20 μs, the effect of proton density becomes more apparent, and ⟨*s*_12_(2τ)⟩^*N*^ shows contributions of proton pairs up to *R* = 15 Å and *r*_12_ = 5 Å (Figure 3b,e). At 100 μs, finally, the effect of large proton numbers in the broader environment of the spin center becomes the dominant factor. While the strongest single contributions are still predicted to be in the region of *R* < 20 Å and *r*_12_ < 4 Å, Figure 3c, the strongly increasing number of protons for large values of *R* and *r*_12_ significantly enhances the effect of pairs with small individual contributions, Figure 3f (see also Figure 2c). Proton pairs from the entire considered region up to *R* = 30 Å and *r*_12_ = 12 Å contribute to the coherence decay. Our analysis illustrates that this outcome is strongly dependent on the density of spins in the environment (see below).

## 4 Analysis of [Cu(dbm)_2_] and (PPh_4_)_2_[Cu(mnt)_2_]

In order to test our analysis for real systems, we have chosen the cases of [Cu­(dbm)_2_] and (PPh_4_)_2_[Cu­(mnt)_2_], diluted in [Pd­(dbm)_2_] and (PPh_4_)_2_[Ni­(mnt)_2_], respectively, as diamagnetic host materials, see Figure 4. For clarity, we will address the two systems as [Cu_*x*_Pd_1–*x*_(dbm)_2_] and (PPh_4_)_2_[Cu_*x*_Ni_1–*x*_(mnt)_2_] in the following, where *x*≪1. These two systems represent two complementary cases, as the former contains protons in the ligand and has no counterions, whereas the latter has proton-free ligands and (proton-bearing) (PPh_4_)^+^ counterions. In both instances, most protons reside in phenyl rings. The coherence times of these two systems were previously measured by pulsed EPR experiments at 7 K on diluted microcrystalline samples (0.001%, i.e., *x* = 10^–5^).^29,51^ For the simulations, we used the experimental crystal structures^64–67^ of the diamagnetic host materials, [Pd­(dbm)_2_] and (PPh_4_)_2_[Ni­(mnt)_2_]. One of the metal ions from the unit cell was selected as the Cu­(II) spin center, and the proton positions in a sphere of radius 35 Å around this spin center defined the spin bath. As the experimental Hahn echo decay was measured on the *g*_⊥_ transition, the average over all magnetic-field orientations in the *xy*-plane (referring to the molecular magnetic axis system) was calculated. This average accounts for the differences of *T*_m_ due to the spatial distribution of the protons; details of the simulation and the dependence of the predicted coherence times on the orientation of the crystal grid relative to the magnetic field are discussed in more detail in Supporting Information Sections S3.1 and S3.2, respectively. The simulated coherence times are 8.31 μs for [Cu_*x*_Pd_1–*x*_(dbm)_2_] (exp. 7.74 μs)^51^ and 10.05 μs for (PPh_4_)_2_[Cu_*x*_Ni_1–*x*_(mnt)_2_] (exp. 9.23 μs),^29^ where the decay curves can be well fitted to a stretched exponential of the form exp­[−(2τ/*T*_m_)^*k*^] with stretch factors of *k* = 2.7 and *k* = 2.8 for the simulated curves of [Cu_*x*_Pd_1–*x*_(dbm)_2_] and (PPh_4_)_2_[Cu_*x*_Ni_1–*x*_(mnt)_2_], respectively (exp. *k* = 2.7 ^51^ and *k* = 2.5 ^29^). The simulated decay curves reproduce very well the trend of the experimental one, and deviations of *T*_m_ and *k* are well below 10% (see Supporting Information Sections S3.3 and S3.4). This observation confirms once again the high accuracy of the APPA model.^51^ We further verified the high-field approximation of the APPA model by comparing the results obtained for the [Cu_*x*_Pd_1–*x*_(dbm)_2_] with the generalized cluster–correlation expansion (CCE) approach developed by Onizhuk and Galli.^61^ Interestingly, only the single-nucleus contributions are affected by variations of the magnetic field, while the nuclear pair (flip-flop) contributions remain nearly unchanged for different field strengths (see Supporting Information Section S3.5).

**Figure 4:**
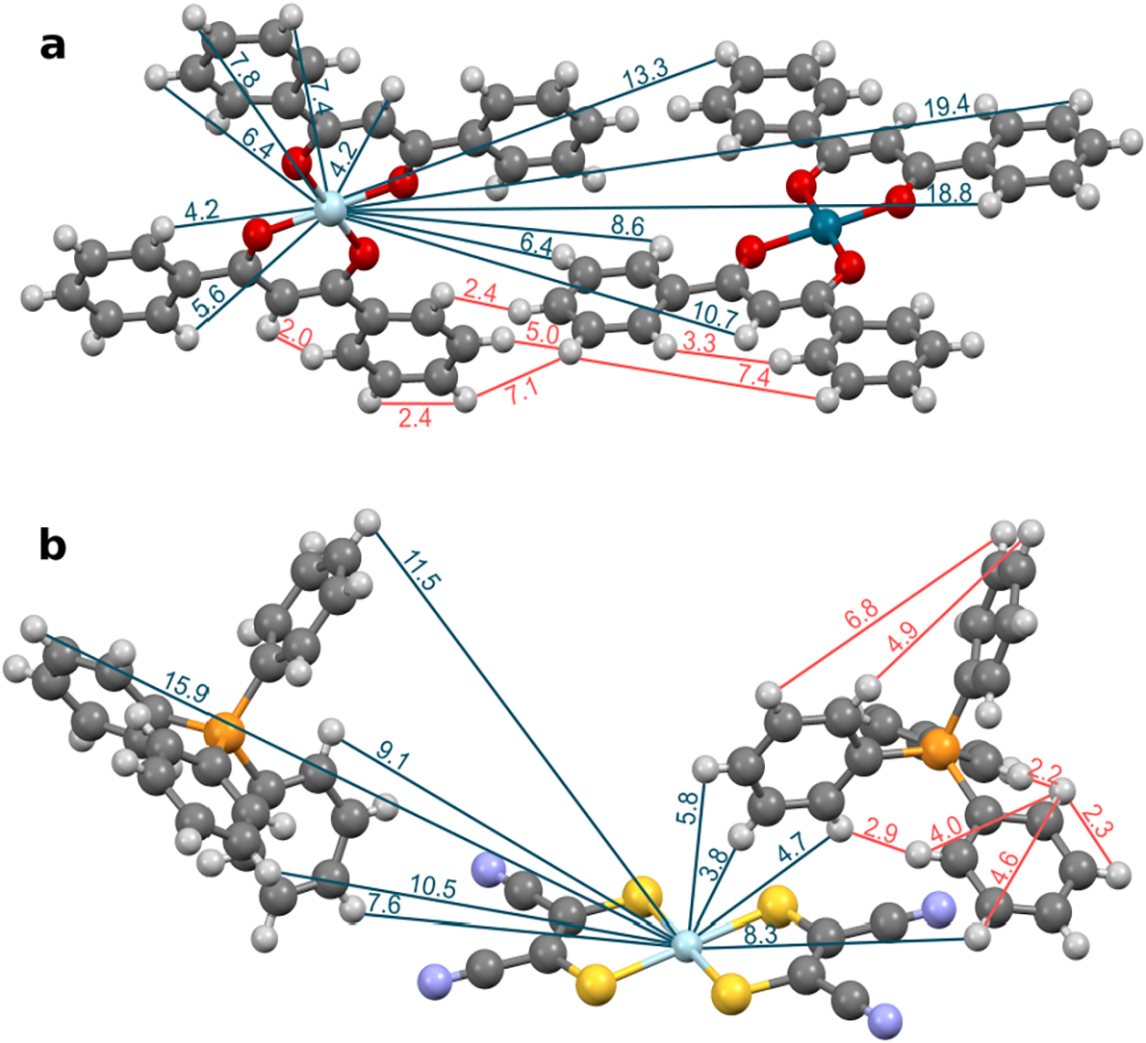
Cutout of the unit cell of (a) [Cu_*x*_Pd_1–*x*_(dbm)_2_] and (b) (PPh_4_)_2_[Cu­(mnt)_2_] showing a selection of distances between the spin center and protons relevant for the decoherence process. Experimental crystal structures from the literature were used.^64–67^ The colors correspond to light cyan = copper, dark blue = palladium, red = oxygen, orange = phosphorus, blue = nitrogen, gray = carbon, and white = hydrogen atoms.

The rather simple structure of the APPA set of equations allows for analyzing these systems by disentangling the decay of the Hahn echo signal into their pair contributions. To this end, we fix the magnetic field at a single orientation that yields a decay time near the average value. For the qualitative analysis, this is of no critical consequence, as the variation of predicted coherence times of the complexes considered here is weak within the *xy*-plane (see Supporting Information section S3.2).

Figure 5a,b shows the modulation depth and modulation frequency of all proton pairs for [Cu_*x*_Pd_1–*x*_(dbm)_2_], averaged over a bin size of 0.25 × 0.25 Å along *R* and *r*_12_; the number of proton pairs for each bin is shown in Figure 5c. The corresponding plots for (PPh_4_)_2_[Cu_*x*_Ni_1–*x*_(mnt)_2_] look very similar (see Supporting Information Section S3.4). These graphs confirm that the main features obtained from the parameter space screening are also present for realistic systems, in particular, the fan-like region of large modulation depth and the decay of the modulation frequency for increasing *R*. Due to the molecular structure and the crystal lattice, there is also a visible fine structure, particularly along the *r*_12_ axis, see Figure 5c.

**Figure 5:**
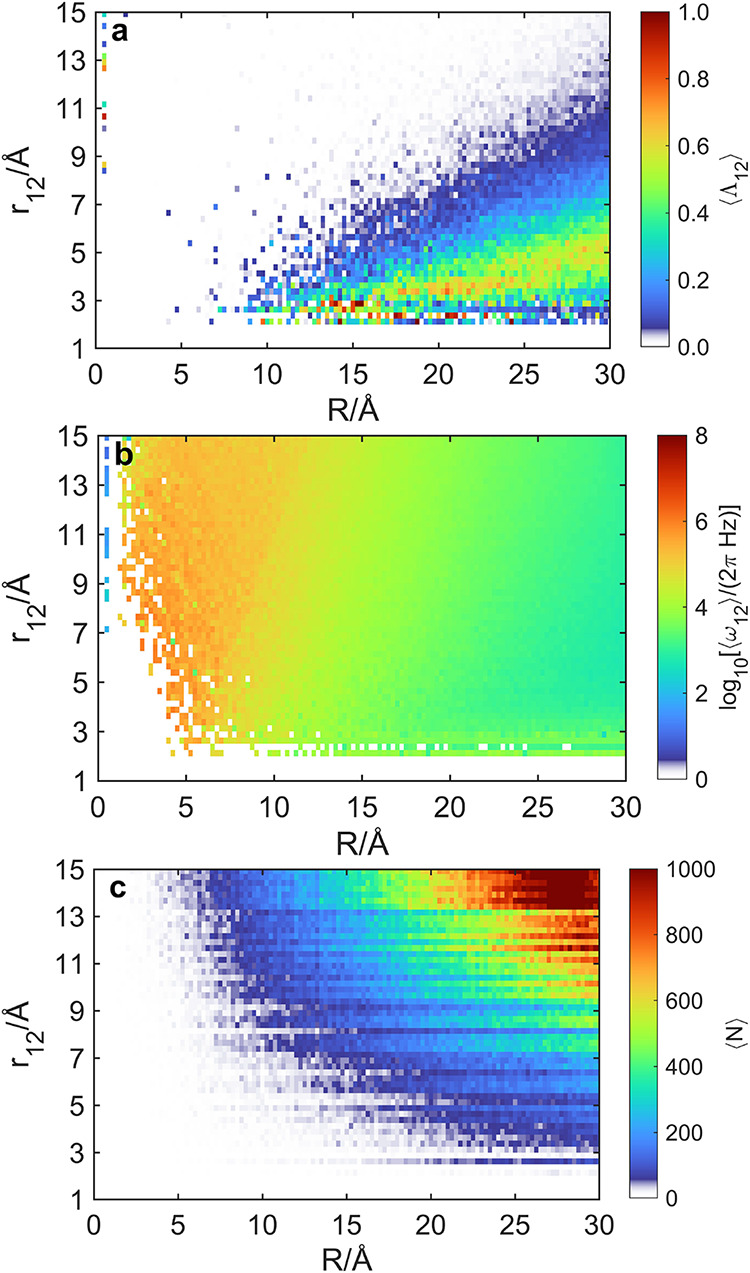
Pair contribution analysis of the spin bath in a [Cu_*x*_Pd_1–*x*_(dbm)_2_] crystal. (a) Modulation depth, (b) modulation frequency, and (c) number of protons. Graphs (a) and (b) show averages over the respective 0.25 Å × 0.25 Å bins, and (c) shows the accumulated number of protons within each bin.

In [Cu_*x*_Pd_1–*x*_(dbm)_2_], the smallest interproton distance is *r*_12_ = 2.0 Å, corresponding to the distance between the proton at the α-carbon of the diketo moiety and the protons in ortho position of either phenyl group, as shown in Figure 4a. The other visible clustering is around 2.3–2.4 Å, which is the distance between vicinal phenyl protons. The first intermolecular proton pairs appear from 2.4 Å onward (between the para and ortho protons of neighboring phenyl groups).

The resulting decay contributions at different delay times are shown in Figure 6 for [Cu_*x*_Pd_1–*x*_(dbm)_2_] and (PPh_4_)_2_[Cu_*x*_Ni_1–*x*_(mnt)_2_]. At 2τ = 8 μs, we are close to the experimental and simulated coherence time at which the Hahn echo intensity has decayed to 1/*e*. At 2τ = 20 μs, the Hahn echo intensity has basically decayed to zero in both cases. We also included 2τ = 100 μs to check the predictions from the parameter space screening for longer times.

**Figure 6:**
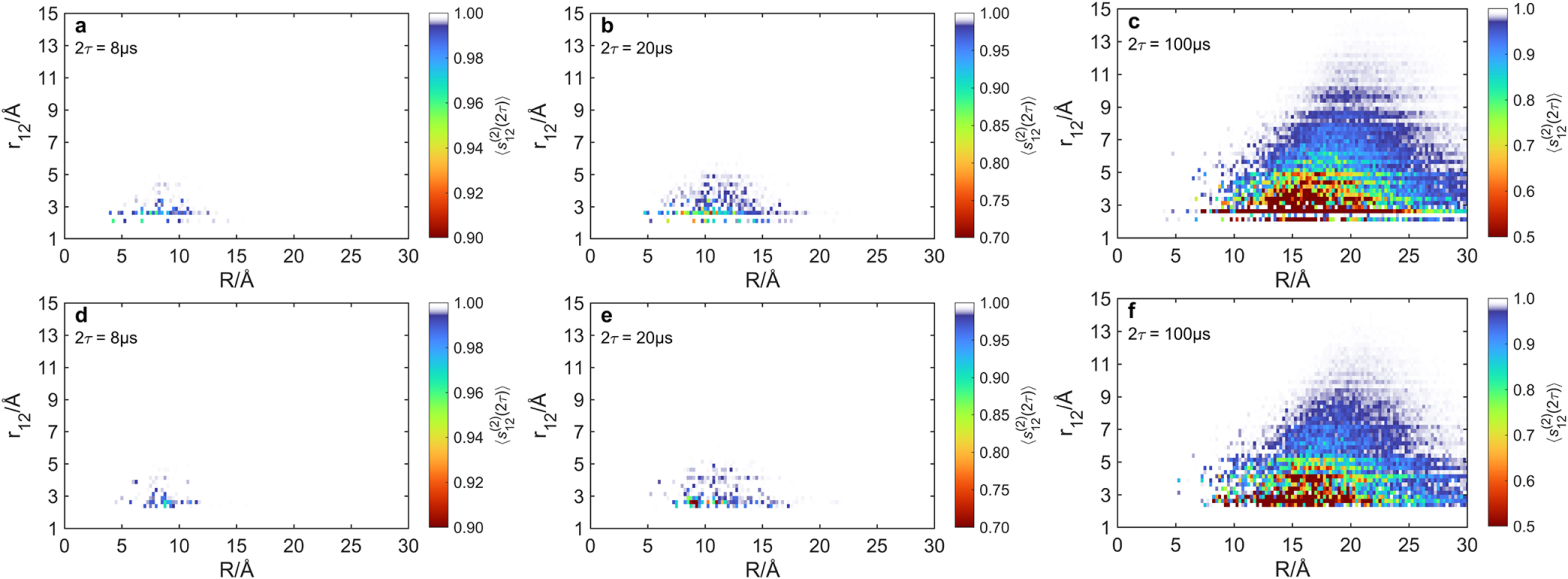
Pair contributions to the Hahn echo decay for different delay times as indicated in the panels, (a–c) [Cu_*x*_Pd_1–*x*_(dbm)_2_] and (d–f) (PPh_4_)_2_[Cu_*x*_Ni_1–*x*_(mnt)_2_]. Note that the color scale has been adapted for the various delay times to enhance visibility.

From our general analysis, it is clear that up to 8 μs, only a rather limited number of proton pairs contribute in both cases, mainly confined to the range 5 < *R* < 15 Å and *r*_12_ < 4 Å. The differences between [Cu_*x*_Pd_1–*x*_(dbm)_2_] and (PPh_4_)_2_[Cu_*x*_Ni_1–*x*_(mnt)_2_] remain rather subtle.

In both cases, the main contributions at 2τ = 8 μs originate from phenyl proton pairs with *r*_12_ = 2.4 Å. For [Cu_*x*_Pd_1–*x*_(dbm)_2_], however, contributions from closer pairs at around 2.0 Å are present, as well. As a second noticeable difference, for (PPh_4_)_2_[Cu_*x*_Ni_1–*x*_(mnt)_2_], most contributions come from pairs with copper–proton distances of *R* > 7 Å, while for [Cu_*x*_Pd_1–*x*_(dbm)_2_], also pairs with smaller values of *R* contribute. In both cases, there are a few contributing pairs with *R* < 5 Å, but these turn out to be of little impact on the overall signal: Removing all pairs with *R* < 5 Å from the product (compare eq 1) increases the signal at 8 μs by only 8%. This also adds a statistical component to the emergence of a spin diffusion barrier: While proton pairs in the direct vicinity of the spin center may have a small nonzero contribution, they are very low in number and will therefore not dominate the time scale of the decay.

At 2τ = 20 μs, the Hahn echo signal has fully decayed for both cases. Interestingly, only a limited range of protons is involved. In fact, restricting the evaluation of the total signal to pairs within the range of 5 < *R* < 15 Å and *r*_12_ < 4 Å gives an intensity that is only 20% too large at 8 μs and is also sufficient to drive the total signal to zero within 20 μs.

The two snapshots for 2τ = 100 μs are rather similar for both systems, Figure 6c,f. The extent of the distribution is well predicted by the parameter space screening, Figure 3f, demonstrating the wide applicability of that approach. In fact, the atomistic simulations confirm that for longer coherence times, a wider range of protons contribute to the decay; in the present cases, protons up to *R* = 30 Å and *r*_12_ = 12 Å are involved.

## 5 Strategies for Coherence Time Improvement

The observations from the previous section show that the short-term contributions to coherence are mainly governed by the nearby protons in the range 2 to 5 Å, in particular, those which are close neighbors, such as the phenyl protons. It is therefore tempting to investigate the effect of removing those. In case of actual compounds, this could be achieved by breaking the pairs, for example, by partial deuteration or chlorination of the phenyl protons. While both of these elements still have spin-bearing nuclei, it is well-known that these couple less strongly to the electron spin, due to their much smaller gyromagnetic ratio. In addition, the quadrupole splitting of these nuclei detunes individual nuclei in nonequivalent positions within the unit cell and further suppresses flip-flop processes.^48^ As their Larmor frequencies also differ from those of the protons, they do not contribute to the proton flip-flop processes. In our simulations, we simply remove protons at deliberate positions.

For both [Cu_*x*_Pd_1–*x*_(dbm)_2_] and (PPh_4_)_2_[Cu_*x*_Ni_1–*x*_(mnt)_2_], we have investigated several substitution patterns at the phenyl group, see Table 1. Of these, substitution of the ortho- and ortho’-protons alone is least efficient (likewise substitution of the para position alone). In these cases, the coherence time is enhanced by approximately 35% (13% for the para position). Clearly, in these cases, a number of close proton pairs are still retained. Substituting the meta and meta’ position effectively removes all proton pairs at *r*_12_ ≈ 2.4 Å, and the simulated coherence time increases by 51% for [Cu_*x*_Pd_1–*x*_(dbm)_2_] and by 67% for (PPh_4_)_2_[Cu_*x*_Ni_1–*x*_(mnt)_2_]. The slightly smaller improvement in the former case is most likely due to the remaining close pairs between the ortho position and the proton at the α-carbon. By substituting ortho, ortho’, and para positions, our simulations predict the strongest improvement. For both molecules, the coherence times more than double and reach nearly 24 μs in the case of the modified (PPh_4_)_2_[Cu_*x*_Ni_1–*x*_(mnt)_2_] system. Similar substitution patterns have been previously considered for a series of V­(IV) compounds,^43,44^ but without clear effect on the coherence times, as these were measured in a glass matrix (fully deuterated o-terphenyl) and at very high field, where additional decoherence mechanisms may play an important role.^44^ We emphasize that the improvement of coherence times does not primarily result from a modification of the molecule bearing the electron spin but from a modification of the surrounding matrix. As a test, we have also run a simulation, where we kept all protons on the central [Cu­(dbm)_2_] and only the [Pd­(dbm)_2_] molecules of the matrix were modified by removing the ortho-, ortho’-, and para-protons. As shown in Table 1, this shortens the coherence time by only 1.7 μs (10%), relative to the simulation in which the central [Cu­(dbm)_2_] was also modified. In view of this result, it becomes clear that modifications of the MSQ alone, like for the previously mentioned work on V­(IV) compounds,^43,44^ will not necessarily lead to significant changes of *T*_m_.

**Table 1:** Simulated Coherence Times (Phase Memory Times *T*_m_) for the Parent Compounds and Different Hypothetical Variants with Substitution of Protons by Spin-Less Atoms

	*T*_m_/μs		
system	sim.^a^	exp.^b^	ρ*_n_*/Å^–3^ ^c^
[Cu_*x*_Pd_1–*x*_(dbm)_2_]	8.31	7.74 ± 0.03	0.0371
o,o’-subst.	11.33		0.0236
m,m’-subst.	12.54		0.0236
o,o’,p-subst.	17.43		0.0169
all-H/o,o’,p-subst.^d^	15.70		0.0169
(PPh_4_)_2_[Cu_*x*_Ni_1–*x*_(mnt)_2_]	10.05	9.23 ± 0.01	0.0339
o,o’-subst.	13.12		0.0204
m,m’-subst.	16.85		0.0204
p-subst.	11.03		0.0271
o,o’,p-subst.	23.48		0.0136

a Simulated phase memory time, determined as the time where the signal has decayed to 1/*e*.

b Experimental phase memory time (fit to stretched exponential) from ref 53 (for [Cu_*x*_Pd_1–*x*_(dbm)_2_]) and ref 29 (for (PPh_4_)_2_[Cu_*x*_Ni_1–*x*_(mnt)_2_]).

c Proton densities.

d Unmodified [Cu­(dbm)_2_] in o,o’,p-substituted [Pd­(dbm)_2_] matrix.

In Table 1, we also report the proton density for the considered cases, which is reduced by up to 60% in the case of the ortho, ortho’, and para substitution. This also has a noticeable effect at longer times, where the contributions from the farther environment are strongly reduced (see Supporting Information Sections S3.3 and S3.4). We note that the coherence times *T*_m_ approximately scale with the inverse of the proton density ρ_n_, which is in line with observations from other work.^48,53^

## 6 Insights

In summary, the analysis of the simulations provides the following insights, which corroborate and extend previous strategies^29,32,37,42–44,46,48^ for improved coherence times:

(a) Protons closer than ≈5 Å to the spin center do not lead to decoherence due to the “diffusion barrier” effect. Although this has been found empirically,^40,41,63^ here, we provide insight into the origin: Our analysis shows that this is in part due to a mismatch of two energy scales: The difference in hyperfine coupling between each of the protons and the central electron spin becomes, in general, much larger than the dipolar coupling of the proton pair, leading to a very small modulation depth. In addition, the effect of the nuclei close to the spin center is small for statistical reasons: the contribution of the few close pairs vanishes in comparison to the *O*(10^4^) remaining pairs.
(b) The main contributions to decoherence result from the close to midrange environment, up to 15 Å from the spin center. The relevant range increases with time, i.e., for very long coherence times approaching the millisecond regime, a considerably larger range around the spin center must be taken into account. In this case, the proton density of the environment will become decisive.
(c) The strongest contributions in the environment come from proton pairs that are close together with distances up to *r*_12_ = 4 Å. The rather close distance yields a significant modulation depth and a sufficiently high modulation frequency for contributing to decoherence on the relevant time scale (typically 1 to 100 μs, corresponding to 1 MHz to 10 kHz).

From these observations, a few strategies for MSQs with improved coherence times can be rationalized. From observation (a), it follows that there is no need to design the MSQ itself proton-free at any price. In the cases discussed in Section 5, the decoherence is mainly driven by proton pairs of the surrounding molecules, while those of the MSQ itself only contribute a little, depending on the size of the MSQ. According to observation (b), the environment has to be designed with the desired time scale in mind. For times up to ∼20 μs, the first coordination sphere around the MSQ must be considered, and bulky and proton-free counterions should be a part of a strategy for reaching coherence times in this range. For reaching coherence times of 100 μs and beyond, the spin density of the environment becomes the decisive factor as the volume around the MSQ that includes nuclear spin pairs contributing to decoherence expands rapidly with time.

Importantly, observation (c) demands an increase in the separation of protons. Partial substitution by nuclei without spin or with a smaller gyromagnetic ratio, like ^2^H or ^35/37^Cl, may achieve this, as in part already used in chlorinated organic radical MSQs based on TTM (tris­(trichlorophenyl)­methyl) units^14^ and in V­(IV) triscatecholate complexes with various elemental substitutions.^43,44^ Nevertheless, it is important to highlight that these strategies are expected to result in a significant lengthening of the coherence times only in case the electronic spins are well-diluted and that there are no other proton spins in the nearby, for example, in the solvent or stabilizing agents. Methyl groups may induce further processes like tunneling, which may significantly shorten coherence times.

## 7 Conclusions

In this work, we have analyzed the coherence decay of molecular spin qubits (MSQs) induced by a nuclear spin bath of protons. Exploiting the rather simple structure of the yet accurate analytic pair product approximation (APPA) for simulating the Hahn echo signal,^50,51,56,57^ we can relate the geometric arrangement of the proton spin pairs and the electron spin center to the underlying physical processes that limit the coherence time of the electron spin state. This parameter space screening and the analysis performed in this work provide clear indications of the geometrical conditions for nuclear spin pairs that lead to maximal decoherence and should therefore be avoided. We also find that the range of contributing nuclei around the spin center increases with the delay time. While at short times up to 10 μs only the local environment within approximately 15 Å contributes significantly, a substantially larger portion of the spin bath surrounding the MSQ becomes relevant on the 100 μs time scale. These results suggest that different strategies may be adopted depending on the intended coherence time scale. For short coherence times, efforts should focus on increasing the separation between proton pairs, for example, by selecting appropriate ligands or via partial elemental substitution. For long coherence times, however, it becomes necessary to reduce the average proton density. These findings provide valuable design guidelines for improving MSQs and their environment, beyond the stringent and often impractical requirement of completely removing all protons. Our approach can also be generalized to other nuclear spins, at the expense of going beyond the APPA equations for nuclei with spins $I>\frac{1}{2}$ . This opens the possibility of exploring heteronuclear interactions between nuclei in the environment having different gyromagnetic ratios in order to identify conditions that lead to increasing coherence times. These developments, in concert with systematic investigations of crystalline host matrices, will be helpful to create a controllable sparse distribution of bath spins and gain further information that will contribute to achieving long coherence times even in the presence of protons and other nuclear spins. This is highly relevant for the realization of quantum architectures where MSQs are placed at specific positions, as, for example, in the case of metal-organic frameworks (MOFs).^68,69^ Moreover, these results are relevant for the realization of crystalline MSQ structures that mimic spin-carrying defects or color centers, with the advantage of having a controllable positioning and a flexible electronic structure.^2,70^

## Supplementary Material



## Data Availability

Additional data are available on ZENODO (10.5281/zenodo.17543342) and in the ESI. The code for the APPA computations on the crystal models is available from https://github.com/KoehnLab/jlcce.

## Notes

(1) Treatment of anisotropic spin centers; (2) details of the parameter space screening; and (3) details on the simulations for [Cu_*x*_Pd_1–*x*_(dbm)_2_] and (PPh_4_)_2_[Cu_*x*_Ni_1–*x*_(mnt)_2_] (PDF)
