# Supplementary material for: Linking Chemical Structure and Coherence Times of Molecular Spin Qubits
Source: JACS Au. 2026 Jun 18;6(7):3927–38. doi: 10.1021/jacsau.6c00489 (PMC13417297; doi:10.1021/jacsau.6c00489)
Supplement: Supplementary file 1 [file au6c00489_si_001.pdf]

Supplementary Information: Linking Chemical Structure and Coherence Times of Molecular Spin Qubits

Sarah Suchaneck,[*,†,¶] Lorenzo Tesi,[*,‡,¶] Joris van Slageren,[*,‡,¶] and Andreas Köhn[*,†,¶]

† *Institute for Theoretical Chemistry, University of Stuttgart, Pfaffenwaldring 55, D-70569 Stuttgart, Germany*

‡ *Institute of Physical Chemistry, University of Stuttgart, Pfaffenwaldring 55, D-70569 Stuttgart, Germany*

¶ *Center for Integrated Quantum Science and Technology (IQST), University of Stuttgart, Pfaffenwaldring 55, D-70569 Stuttgart, Germany*

E-mail: suchaneck@theochem.uni-stuttgart.de; lorenzo.tesi@ipc.uni-stuttgart.de; slageren@ipc.uni-stuttgart.de; koehn@theochem.uni-stuttgart.de

Contents

S1 Treatment of anisotropic spin-centres within the high-field approximation S2

S2 Details of the parameter space screening S4

S2.1 Coordinate system S4

S2.2 Simulation details S5

S2.3 Screening of the Jacobi angle α S5

S2.4 Screening of the magnetic field direction S9

S2.5 Averaging over Jacobi angle and magnetic field orientation S12

S2.6 Slope of maximum for modulation depth S15

S3 Computational details of the investigation of $[Cu_xPd_{1-x}(dbm)_2]$ and $(PPh_4)_2[Cu_xNi_{1-x}(mnt)_2]$ S18

S3.1 Implementation of the analytic pair product approximation in Julia S18

S3.2 Dependence on the magnetic field orientation S19

S3.3 Analysis of the nuclear pair contributions of $[Cu_xPd_{1-x}(dbm)_2]$ S22

S3.4 Analysis of the nuclear pair contributions of $(PPh_4)_2[Cu_xNi_{1-x}(mnt)_2]$ S26

S3.5 Test of the high-field approximation S30

References S31

S1 Treatment of anisotropic spin-centres within the high-field approximation

The standard formulation of the analytic pair product approximation only considers an electronic central spin with isotropic g tensor. Here, it is shown how anisotropic g tensors can be easily treated within the high-field approximation. The general spin Hamiltonian for

the Zeeman term is

$$\hat{H}_{\mathrm{Z}} = \frac{\mu_{\mathrm{B}}}{\hbar}\mathbf{B}_0^T\mathbf{g}\,\hat{\mathbf{S}} \tag{S1}$$

where μ_{B} and $\hbar$ are the Bohr magneton and reduced Planck constant, respectively, $\mathbf{B}_0$ the applied (static) magnetic field, and $\hat{\mathbf{S}}$ the vector operator of the electron spin. The g tensor can be written in terms of its main axis components, collected on the diagonal of the matrix $\mathbf{g}_d$ and a rotation matrix $\mathbf{R}$ describing the orientation of the magnetic axes of the spin centre relative to any other coordinate system:

$$\mathbf{g} = \mathbf{R}\mathbf{g}_d\mathbf{R}^T\,. \tag{S2}$$

In the high-field approximation, the direction of the applied magnetic field defines the quantization axis of the spin. This direction will be labelled as 'z' and only the spin component along this direction will be considered (and denoted '$\hat{S}_z$' hereafter). Letting the unit vector $\mathbf{n}$ denoting the direction of the magnetic field, we can write

$$\mathbf{B} = B_0\mathbf{n}\,, \qquad \hat{\mathbf{S}} = \mathbf{n}\hat{S}_z \tag{S3}$$

Inserting this into the general Zeeman expression, eq. S1, we obtain

$$\hat{H}_{\mathrm{Z}} = \frac{\mu_{\mathrm{B}}}{\hbar}B_0(\mathbf{n}^T\mathbf{R}\mathbf{g}_d\mathbf{R}^T\mathbf{n})\hat{S}_z = \frac{\mu_{\mathrm{B}}}{\hbar}B_0 g_{\mathrm{eff}}\hat{S}_z \tag{S4}$$

from which we deduce the expression for the effective g value

$$g_{\mathrm{eff}} = (\mathbf{n}^T\mathbf{R}\mathbf{g}_d\mathbf{R}^T\mathbf{n}) \tag{S5}$$

This effective g value is also used in the expression for the hyperfine coupling, as shown in the main text.

S2 Details of the parameter space screening

S2.1 Coordinate system

For a systematic screening of the geometric parameters that enter the expression for the pair contribution to the Hahn echo decay within the APPA, we have introduced a set of 5 parameters, see also Fig. 1 of the main text. The Jacobi coordinates R, r_{12}, and α describe the relative arrangement of the three spins. We use a convenient reference coordinate system to express these coordinates in Cartesian coordinates and place the nuclei in the xy-plane with R (connecting the electron spin centre and the midpoint of the nuclear spins) oriented along the x axis and the centre of the distance vector between the nuclei, r_{12}, at the origin. The Cartesian representation of these vectors is then:

$$\mathbf{R} = \begin{pmatrix} R \\ 0 \\ 0 \end{pmatrix}, \qquad \mathbf{r}_{12} = \begin{pmatrix} r_{12} \cdot \cos\alpha \\ r_{12} \cdot \sin\alpha \\ 0 \end{pmatrix}. \tag{S6}$$

From this, the distance vectors between the electron spin centre and each nucleus can be computed:

$$\mathbf{r}_1 = \mathbf{R} - \frac{1}{2}\mathbf{r}_{12}, \tag{S7}$$

$$\mathbf{r}_2 = \mathbf{R} + \frac{1}{2}\mathbf{r}_{12}.. \tag{S8}$$

The arrangement of the plane defined by the nuclei relative to the magnetic field $\mathbf{B}_0$ can be parametrised by the polar angles θ and ϕ, such that the normal vector of the magnetic field

direction becomes:

$$\mathbf{n} = \begin{pmatrix} \cos\phi \sin\theta \\ \sin\phi \sin\theta \\ \cos\theta \end{pmatrix} \tag{S9}$$

The relevant distances for the dipolar interactions can be computed as the norms of these vectors and the angles of the magnetic field with any of the three vectors $\mathbf{r}_i \in \{\mathbf{r}_1, \mathbf{r}_2, \mathbf{r}_{12}\}$ can be computed as:

$$\theta_i = \operatorname{atan}\left(\frac{|\mathbf{r}_i \times \mathbf{n}|}{\mathbf{r}_i \cdot \mathbf{n}}\right). \tag{S10}$$

S2.2 Simulation details

The parameter space screening simulations were performed with a code written in Julia (see `https://julialang.org`), see also section S3.1. For these simulations, the following g factors for the electron and the protons where used: $g_\mathrm{e} = 2.002$ and $g_\mathrm{n} = 5.586$. The R and r_{12} parameters were scanned over a range of 1 to 35 Å with a step size of 0.25 Å. The other parameters where either fixed to certain values, or an averaging procedure was applied, as detailed below. A collection of scripts for reproducing the data of this work is deposited along with the raw data on ZENODO (`https://doi.org/10.5281/zenodo.17543342`).

S2.3 Screening of the Jacobi angle α

This section contains a number of screening tests for the influence of the Jacobi angle α on the Hahn echo decay pair contribution. For the screening, the magnetic field is kept perpendicular to the spin system ($\theta = 0$, ϕ arbitrary). Fig. S1 shows R, r_{12} scans of the modulation depth Λ_{12}, the nuclear zero-quantum frequency ω_{12}, and the Hahn echo signal for varying angles α. Figs. S2 and S3 show scans of Λ_{12} and ω_{12} for varying α (0 to 360°, increments of 2°) and fixed values of R and r_{12}.

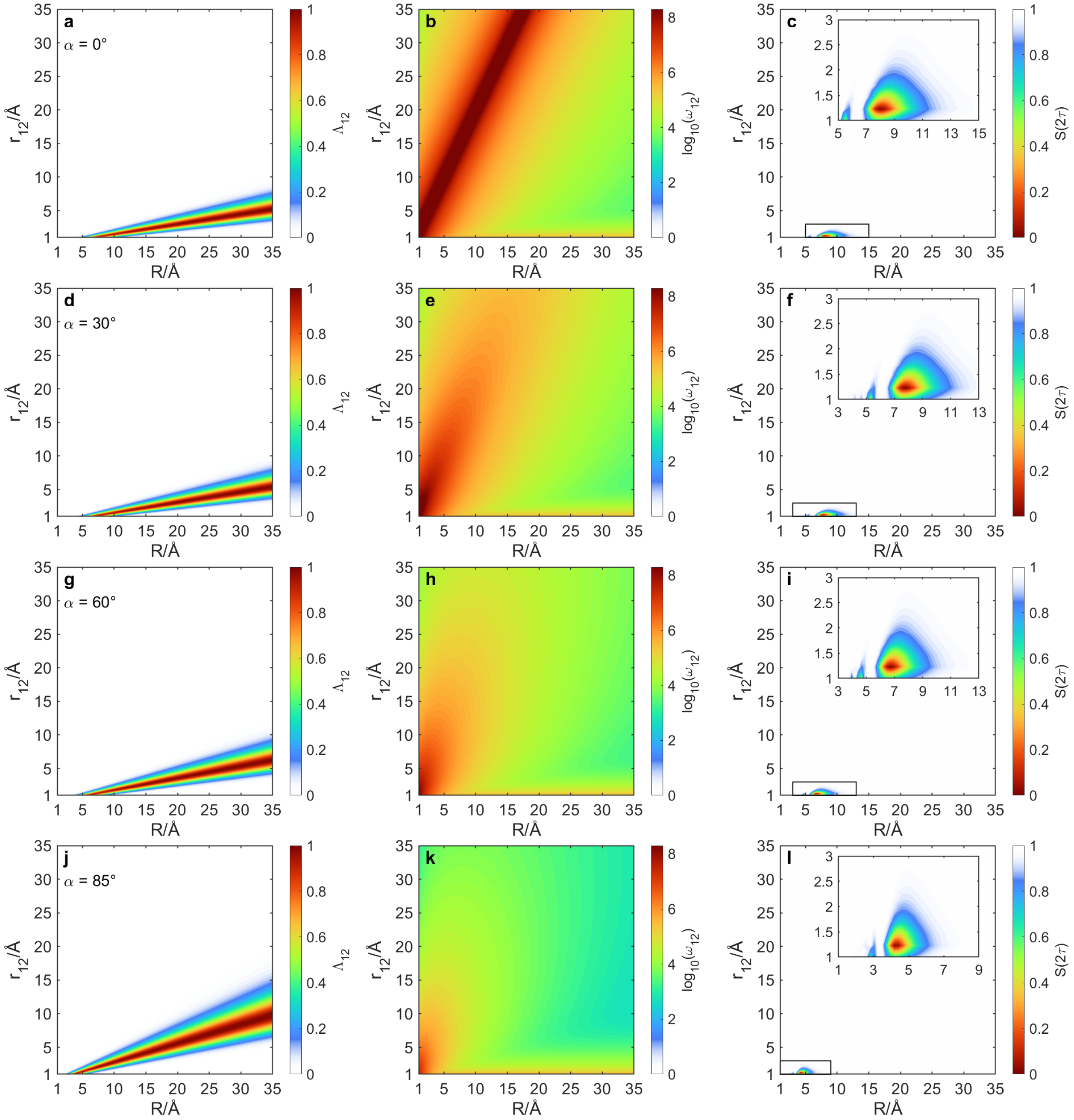

Figure S1: Parameter space scans for different fixed values of α. The magnetic field was set perpendicular to the spin system $(\theta, \phi = 0)$. Left column: modulation depth, middle column decadic logarithm of nuclear zero-quantum frequency; right column: Hahn echo signal at $2\tau = 30$ µs.

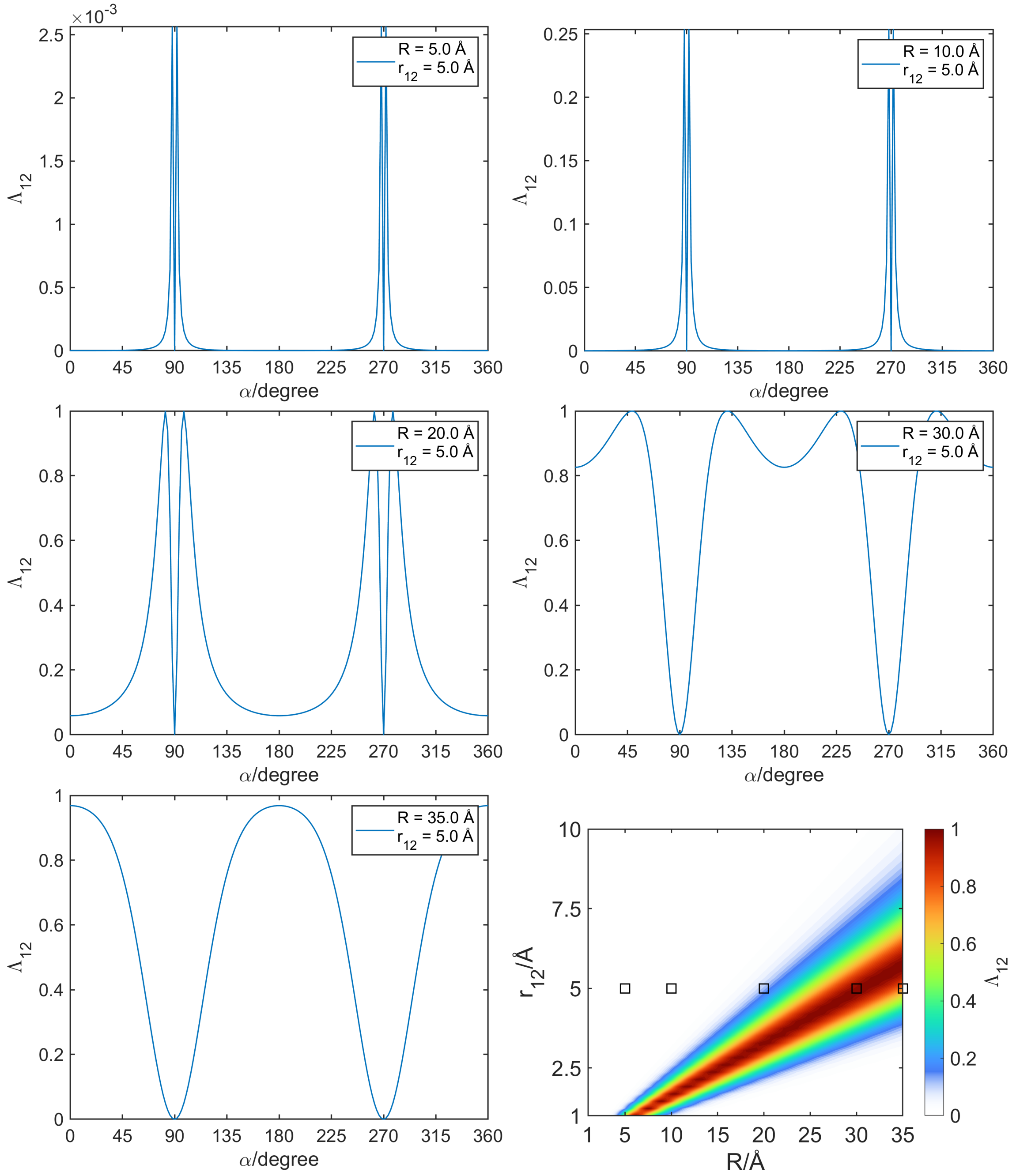

Figure S2: Modulation depth as a function of the Jacobi angle α for different fixed combinations of the distances R and r_{12}.

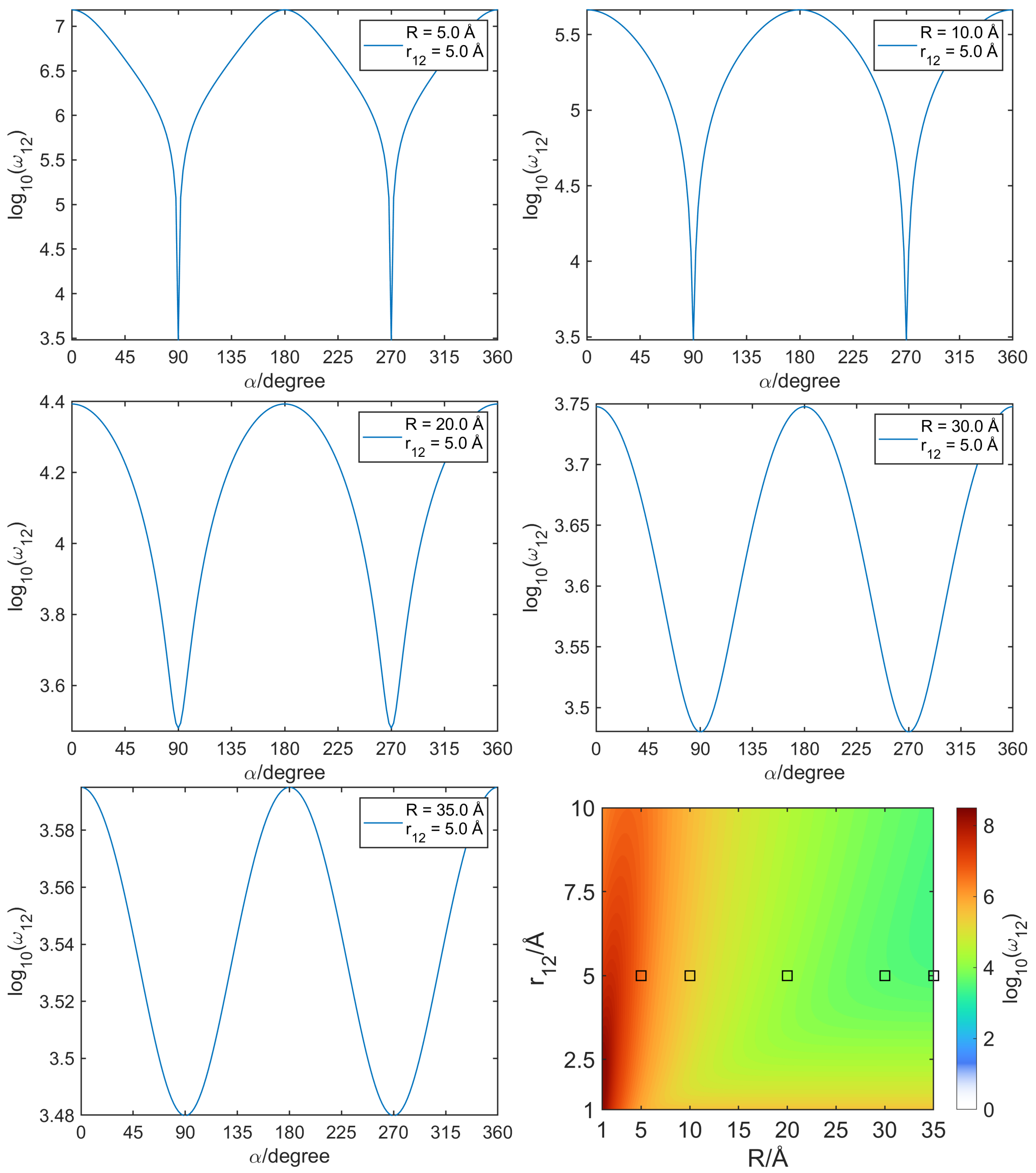

Figure S3: Decadic logarithm of the nuclear zero-quantum frequency as a function of the Jacobi angle α for different fixed combinations of the distances R and r_{12}.

S2.4 Screening of the magnetic field direction

This section contains a number of screening tests for the influence of the relative magnetic field orientation on the Hahn echo decay pair contribution. For the screening, the Jacobi angle α was set to 45°.

Figs. S4 and S5 show (R, r_{12}) scans of Λ_{12}, ω_{12}, and the pair contribution to the Hahn echo decay for varying magnetic field orientations (θ, ϕ). Fig. S6 shows scans of Λ_{12} and ω_{12} for varying magnetic field direction (θ form 0 to 180° and ϕ from 0 to 360° with step sizes of 2°) and fixed values of R and r_{12}.

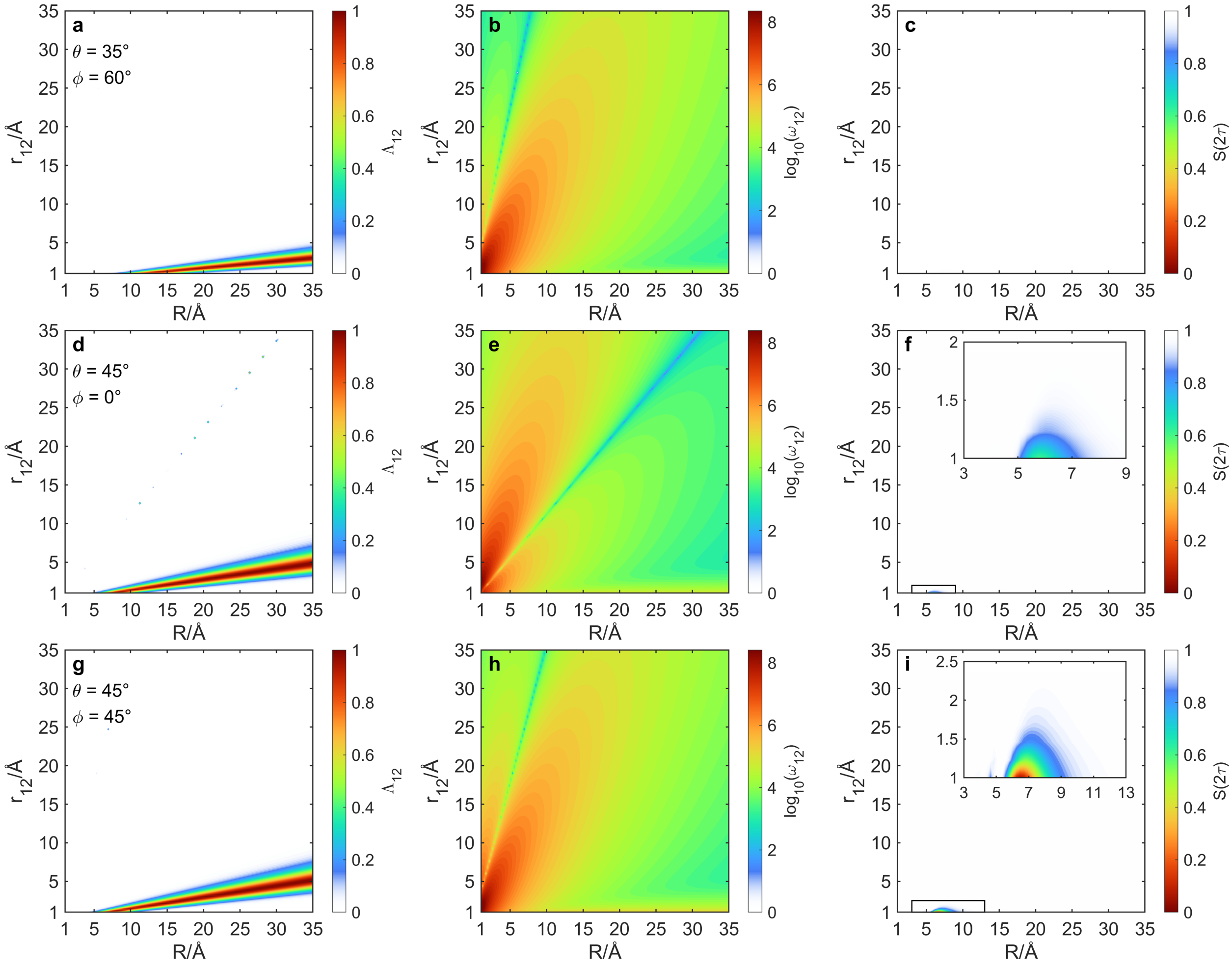

Figure S4: Parameter space scans for different fixed values of the magnetic field direction; Left column: modulation depth, middle column decadic logarithm of nuclear zero-quantum frequency; right column: pair contribution at $2\tau = 30$ μs.

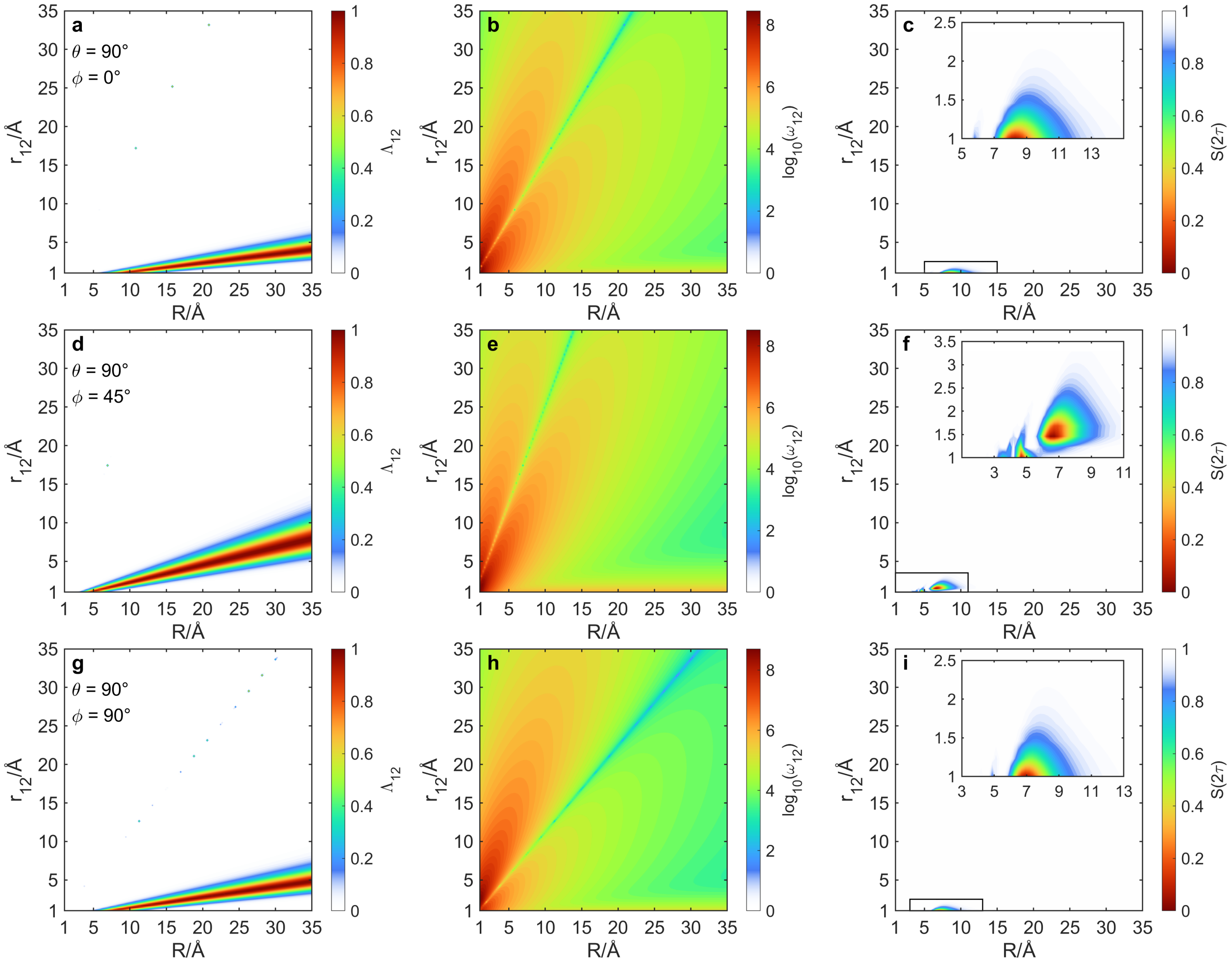

Figure S5: Parameter space scans (continued) for different fixed values of the magnetic field direction; Left column: modulation depth, middle column decadic logarithm of nuclear zero-quantum frequency; right column: pair contribution at $2\tau = 30$ µs.

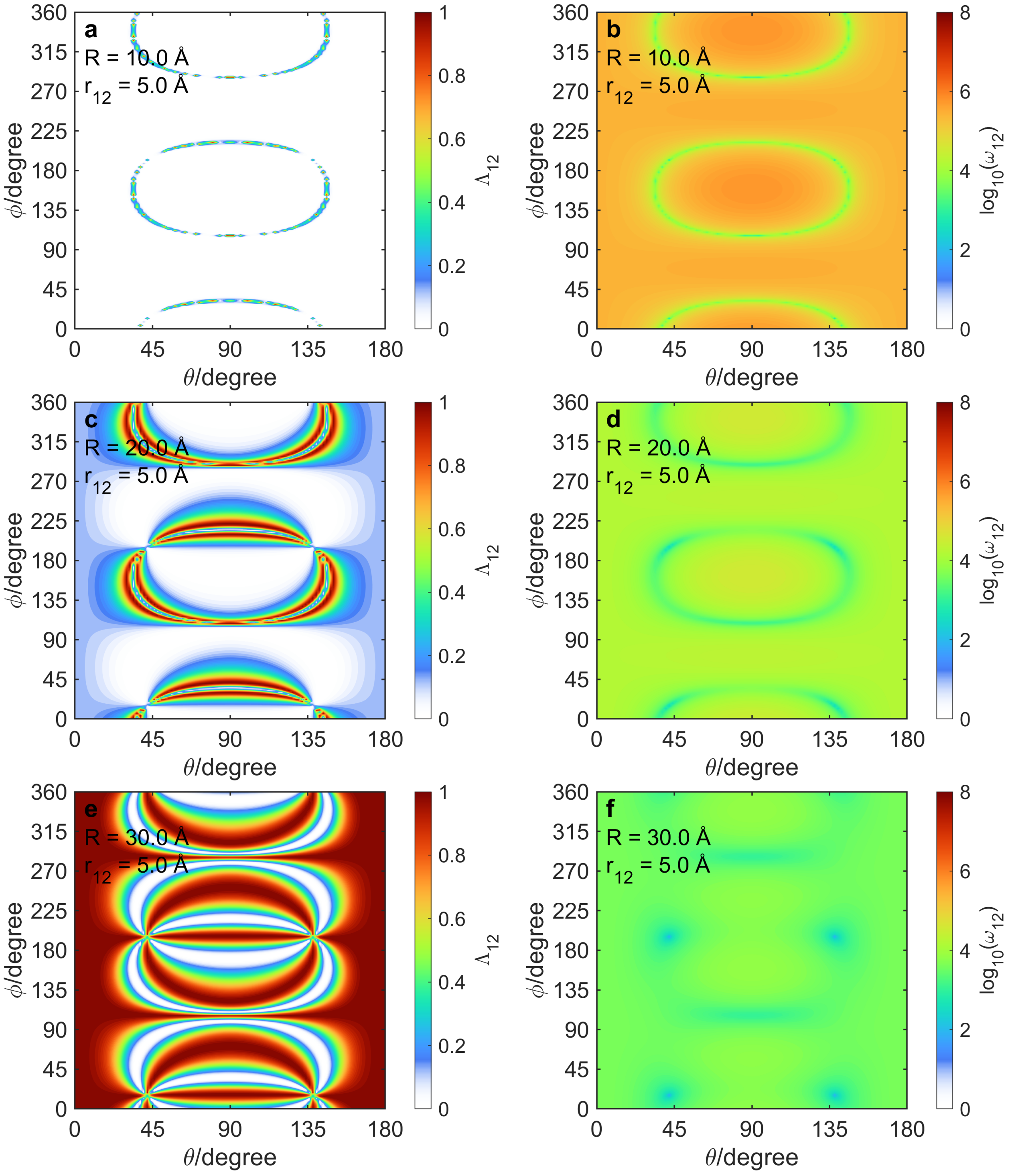

Figure S6: Parameter space dependence on the magnetic field orientation for fixed distances. The magnetic field is rotated by the angles θ and ϕ; Left column: modulation depth, middle column decadic logarithm of nuclear zero-quantum frequency.

S2.5 Averaging over Jacobi angle and magnetic field orientation

The observations from the previous sections suggest that the dependence of Λ_{12} and ω_{12} is very similar for most Jacobi angles and orientations relative to the magnetic field. This prompted us to compute averages over these angles. To this end, we performed numerical integrations: (i) We averaged over the Jacobi angle α via a trapezoidal rule quadrature, and (ii) over magnetic field orientation $\mathbf{B}_0$ via Lebedev quadrature. Both quadratures were implemented in Julia using the NumericalIntegration (`https://juliapackages.com/p/numericalintegration`) and Lebedev (`https://juliapackages.com/p/lebedev`) packages. For the integration over α we also compared to an adaptive quadrature (Gauss-Konrod quadrature) as implemented in the package QuadGK (`https://juliamath.github.io/QuadGK.jl`).

The integration over $\alpha \in [0, 2\pi]$ was performed using a grid of 360 points. An example is shown in Fig. S7 for fixed magnetic field orientation. Comparison to an accurate adaptive quadrature confirms a good quality of integration by the trapezoidal rule, which is sufficient for the purposes of this work. We are aware that very sharp features as seen for some of the α scans in Figs. S2 and S3 may not be faithfully integrated by the fixed quadrature scheme. However, this likely only happens for cases in which the integral will be dominated by the values outside the problematic regions. Furthermore, all integrands are bound, which further reduces the chance of severe integration errors.

The orientation averaging over $\mathbf{B}_0$ was performed by a Lebedev quadrature of order 53 (974 points). In Fig. S8 we test the quality of integration grids of increasing order (for fixed α) which shows that the chosen order is sufficient for our purposes.

The final averaging was then performed in two steps: For each point (R, r_{12}) we iterate over all Lebedev grid points (θ_i, ϕ_i) and compute the average over α by the trapezoidal rule. The values for all Lebedev grid points are then summed up, employing the appropriate weights given by the Lebedev scheme.

In case of the pair contribution to the Hahn-echo signal, $s_{12}(2\tau)$, the average was actually

taken over $1-s_{12}(2\tau)$, as small deviations from 0.0 are more accurately represented in double precision arithmetic than small deviations from 1.0, leading to more stable results. The final average value was then computed as $\langle s_{12}(2\tau)\rangle = 1 - \langle(1 - s_{12}(2\tau)\rangle$.

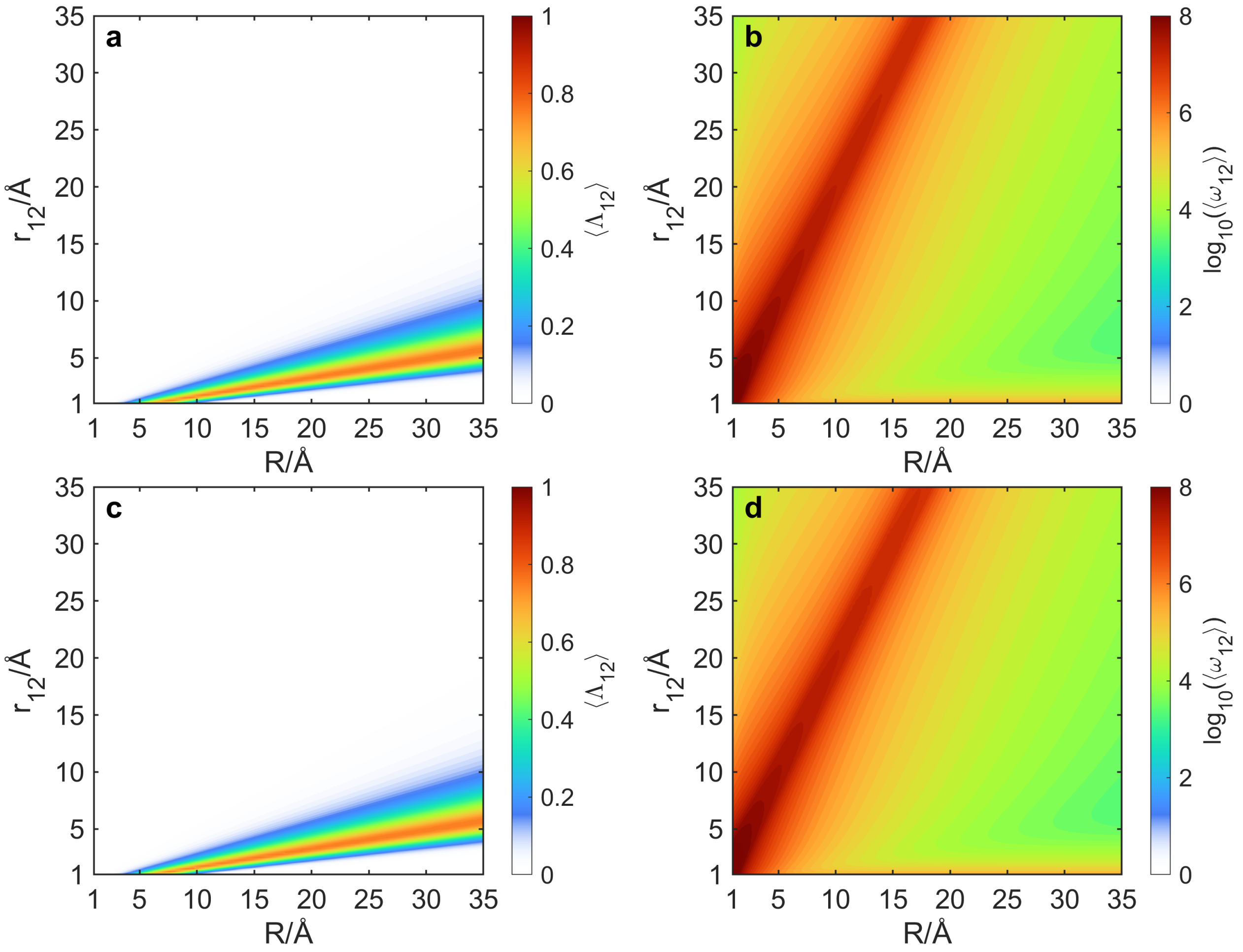

Figure S7: Averaging over α for fixed magnetic field orientation ($\theta = 0$, ϕ arbitrary): left column: Averaged modulation depth, right column: Decadic logarithm of the averaged nuclear zero-quantum frequency as a function of the distances R and r_{12}. (a)+(b): trapezoidal rule quadrature, (c)+(d): Gauss-Kronrod quadrature.

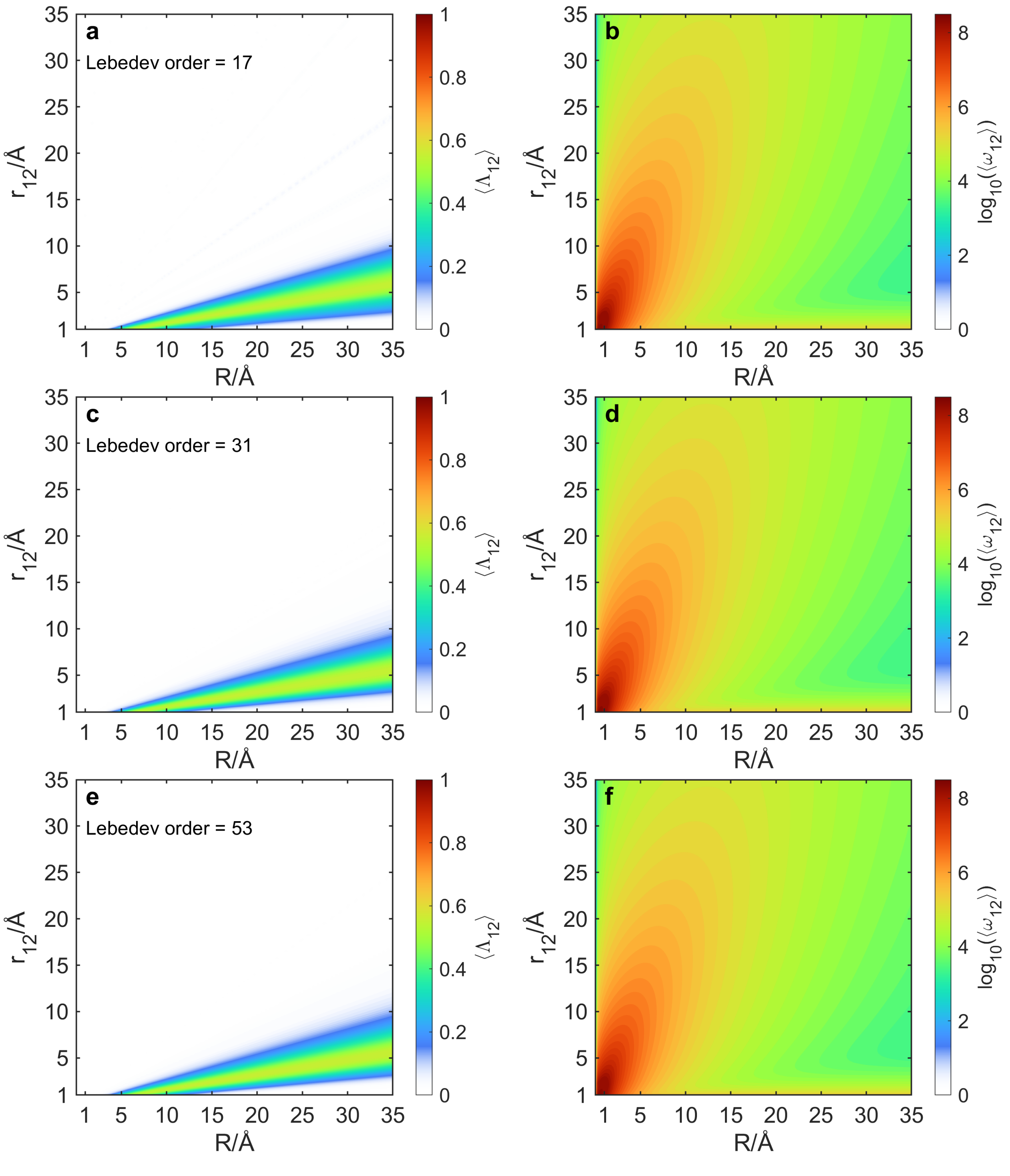

Figure S8: Averaging over $\mathbf{B}_0$ for fixed $\alpha = 45°$. Left column: Averaged modulation depth; right column averaged decadic logarithm of the nuclear zero-quantum frequency. First row: Lebedev quadrature of order 17 (110 points), middle row: Lebedev quadrature of order 31 (350 points), bottom row: Lebendev quadrature of order 53 (974 points).

S2.6 Slope of maximum for modulation depth

The plots of Λ_{12} as a function of R and r_{12}, like those in Fig. S1, show a maximum with a linear relation between R and r_{12}. In the following, we develop an approximate expression for this slope.

The maximum of Λ_{12} is subject to the condition

$$\frac{|A_1 - A_2|}{|b_{12}|} \stackrel{!}{=} 1 \tag{S11}$$

The numerator depends on the difference in hyperfine coupling of the electron spin centre with either nucleus 1 or nucleus 2. For fixed α and $\mathbf{B}_0$ the differences of the distances r_1 and r_2 (describing the distance of the respective nucleus to the electron spin centre) will cause non-zero values. We use Jacobi coordinates and express r_1 with the help of the cosine theorem

$$r_1 = \sqrt{R^2 - Rr_{12}\cos\alpha + r_{12}^2/4} \tag{S12}$$

$$= R\sqrt{1 - (r_{12}/R)\cos\alpha + r_{12}^2/(4R^2)} \tag{S13}$$

$$= R - \frac{r_{12}}{2}\cos(\alpha) + o\Big((r_{12}/R)^2\Big) \tag{S14}$$

where the last approximation is valid for $R \gg r_{12}$. Analogously we express the other distance as

$$r_2 = \sqrt{R^2 - (Rr_{12})\cos(\pi - \alpha) + r_{12}^2/4} \tag{S15}$$

$$= R + \frac{r_{12}}{2}\cos(\alpha) + o\Big((r_{12}/R)^2\Big) \tag{S16}$$

Expanding the dipolar hyperfine coupling interaction at a distance $R + \delta r$ to first order and

inserting for δr the above expansions, eqs. S14 or S16, we get

$$\frac{1}{r_1^3} = \frac{1}{R^3} + \frac{3}{R^4}\frac{r_{12}}{2}\cos\alpha + \dots \tag{S17}$$

$$\frac{1}{r_2^3} = \frac{1}{R^3} - \frac{3}{R^4}\frac{r_{12}}{2}\cos\alpha + \dots \tag{S18}$$

Thus, the difference in hyperfine coupling can be rewritten as

$$A_1 - A_2 \approx \gamma_S\gamma_n\hbar\Big(1 - 3\cos^2\bar{\theta}\Big)\frac{3r_{12}\cos(\alpha)}{R^4} \tag{S19}$$

with assuming an average angle to the magnetic field $\bar{\theta}$. The expression for b_{12} is given by

$$b_{12} = -\gamma_n^2\hbar\frac{1 - 3\cos^2(\theta_{12})}{r_{12}^3}\,. \tag{S20}$$

Inserting these expressions into the matching condition eq. S11 leads to

$$\frac{r_{12}}{R} = C\left|\frac{\gamma_n}{\gamma_S}\right|^{\frac{1}{4}}, \quad \text{with} \quad C = \left|\frac{1}{3\cos\alpha}\frac{1 - 3\cos^2\theta_{12}}{1 - 3\cos^2\bar{\theta}}\right|^{\frac{1}{4}} \tag{S21}$$

Mainly, the expression emphasizes the role of the ratio of gyromagnetic factors γ_n/γ_S for the matching condition.

For the largest part of the parameter space, the prefactor C only weakly depends on the remaining parameters (mainly by virtue of taking the fourth root in this expression). For the α dependence we have $1/3 \leq 1/|3\cos\alpha| < \infty$, where the divergence only appears for α very near to 90° and 270° (note that at these angles, the difference in hyperfine coupling, and thus Λ_{12} becomes small). In Figure S9 we show that the expression is very accurate, when the magnetic field dependence is trivial (perpendicular field, all $\cos\theta$ factors are unity).

The magnetic field dependence is a bit more complicated. The factor $0 \leq |1-3\cos^2\theta| < 2$ is bound, however, so the numerator of eq. (S21) is bound, too. The denominator can also become zero, which requires more attention. However, a zero appears in this case only if

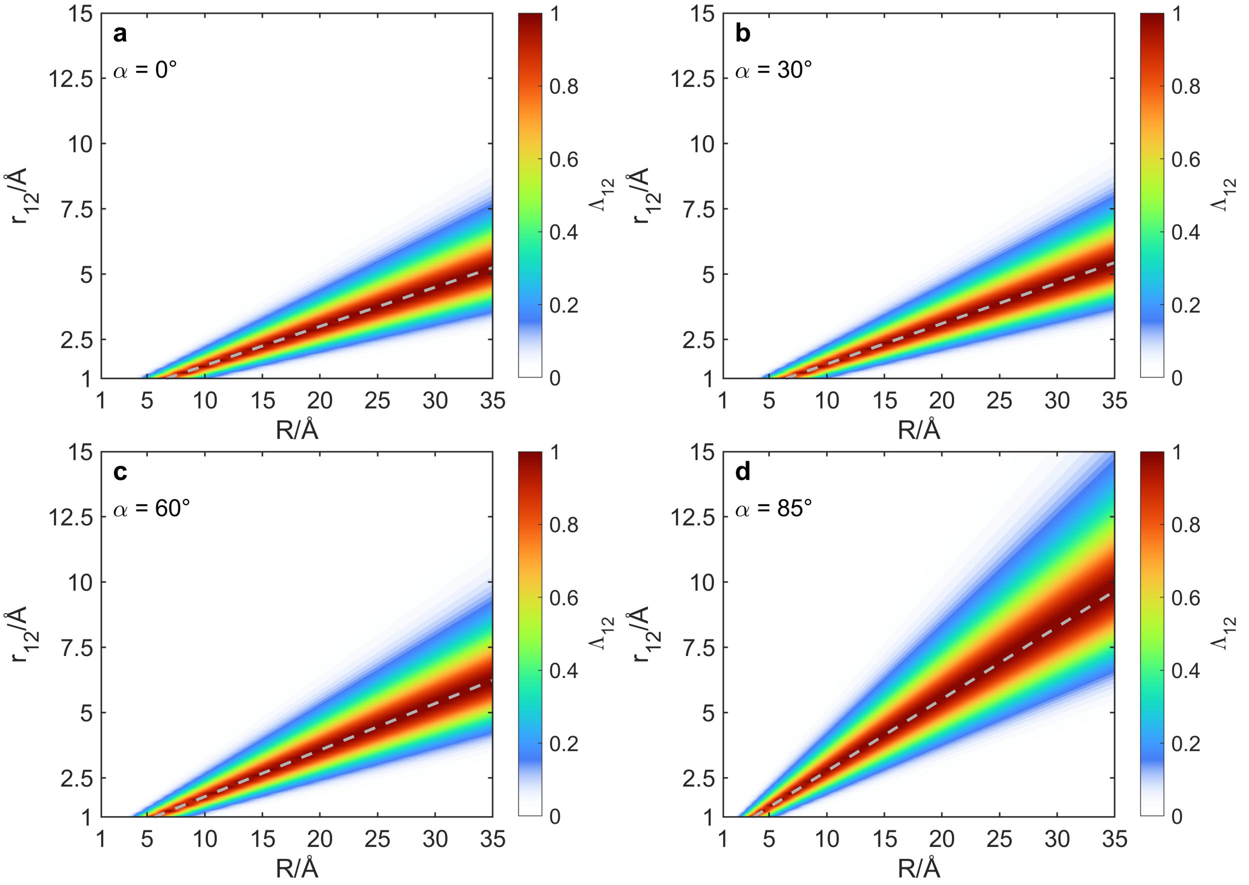

Figure S9: Demonstration of the validity of eq. S21 (dashed line) for perpendicular $\mathbf{B}_0$ and different values of α: (a) $\alpha = 0°$, (b) $\alpha = 30°$, (c) $\alpha = 60°$, and (d) $\alpha = 85°$.

we approach the magic angle where dipolar interactions will be suppressed, leading to a vanishing contribution to the Hahn-echo signal in any way.

S3 Computational details of the investigation of $[Cu_xPd_{1-x}(dbm)_2]$ and $(PPh_4)_2[Cu_xNi_{1-x}(mnt)_2]$

S3.1 Implementation of the analytic pair product approximation in Julia

The analytic pair product approximation was implemented as part of a small Julia library for treating spin dynamics in the cluster-correlation expansion framework. The implementation is based on the theory outlined in Refs. 1–3 and a previous MatLab implementation by Lenz and co-workers.[4] The expression are only valid for spin-$\frac{1}{2}$ centres, as an extension of previous work our implementation also allows to consider anisotropic electron spin centres, as outlined in Section S1.

The following general workflow was implemented:

1. Read in crystal structure data by the `AtomsIO` package (`https://juliapackages.com/p/atomsio`, the package supports in particular CIF and PDB formats)

2. Pick one of the nuclei in the crystal as the spin centre; define g tensor, determine orientation of parallel and perpendicular component within crystal framework (determined from geometry, currently only for square planar complexes).

3. Collect positions of protons in a spheres of radius R around the spin centre (default in this work: 35 Å)

4. Determine hyperfine couplings A_k for all spin-nucleus pairs within the selected set of nuclei

5. Determine a list of pairs of nuclei (maximum distance r_{12}, default in this work: 15 Å)

6. Loop over all pairs, evaluate pair contribution for a predefined set of times and increment the total signal for this set of times.

For $[Cu_xPd_{1-x}(dbm)_2]$, the g factor $[2.051, 2.051, 2.258]$ was used, in case of $(PPh_4)_2[Cu_xNi_{1-x}(mnt)_2]$, the g factor was $[2.0227, 2.0227, 2.0925]$. The z component is in both cases oriented perpendicular to the molecular plane. In these crystal structures, all Cu positions are symmetry equivalent, by default the first occurrence in the definition of the unit cell is picked. All other Cu nuclei are assumed to be substituted by a diamagnetic ion.

S3.2 Dependence on the magnetic field orientation

The result of the simulation depends on the relative orientation of the external magnetic field and the crystal lattice. In practice, measurements are mostly performed on powder samples, which requires an appropriate averaging of the simulation results. In this respect, it must also be considered that the experimental setting allows to focus on a certain resonance frequency, which in the present cases corresponds to the perpendicular component of the g tensor. We can therefore assume that mainly those Cu(II) ions in the sample respond to the $\pi/2$ and π pulses, which have the xy plane of their magnetic axis system oriented along the field, see below. Therefore, we only need to average over all field directions in this plane, which can be determined from the crystal structure data. In both cases, $[Cu_xPd_{1-x}(dbm)_2]$ and $(PPh_4)_2[Cu_xNi_{1-x}(mnt)_2]$, this plane coincides with the plane given by the square-planar coordination around the Cu(II) ion.

In Fig. S10 and Fig. S11, we show the result of a comprehensive scan of simulated T_m times of $[Cu_xPd_{1-x}(dbm)_2]$ and $(PPh_4)_2[Cu_xNi_{1-x}(mnt)_2]$ for all possible magnetic field directions (10° increments). The scan angles θ and ϕ refer to the magnetic axis system of the considered spin centre.

We find a variation of memory times between 7.5 and 11 μs for $[Cu_xPd_{1-x}(dbm)_2]$, and 8.5 and 11 μs for $(PPh_4)_2[Cu_xNi_{1-x}(mnt)_2]$. These variations are mainly dictated by the anisotropy of the spin bath. To compare with the experimental T_m times, we determined the averaged value of T_m along the perpendicular orientation of the magnetic anisotropy. This was done for both $[Cu_xPd_{1-x}(dbm)_2]$ and $(PPh_4)_2[Cu_xNi_{1-x}(mnt)_2]$ by sampling ϕ values

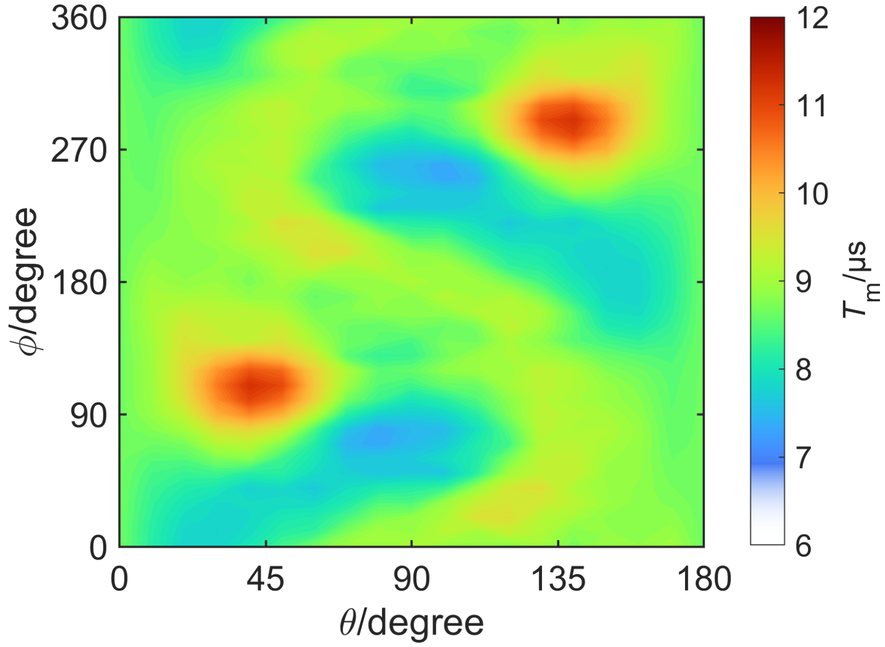

Figure S10: Coherence time T_m of $[Cu_xPd_{1-x}(dbm)_2]$ as a function of the magnetic field orientation. The magnetic field vector was rotated by the polar angle $\theta = 0° \ldots 180°$ and the azimuthal angle $\phi = 0° \ldots 360°$ in $10°$ steps.

with $1°$ increments at fixed $\theta = 90°$.

We also checked the influence of the anisotropy of the spin centre. To this end, we focussed the maximum and minimum values of T_m for $[Cu_xPd_{1-x}(dbm)_2]$ in the entire orientation map (at $\theta = 40°/\phi = 100°$ and $\theta = 80°/\phi = 80°$, respectively, compare to Fig. S10). For these field orientations we recomputed the T_m times assuming an isotropic g-value equal to either 2.051 or 2.258, i.e. the values of $g_\perp$ or $g_\parallel$, respectively. We obtain T_m times of 11.14 µs for $g_\perp$ and 10.84 µs for $g_\parallel$, in the most favourable orientation of the proton atoms. For the least favourable orientation, these times are 7.36 µs for $g_\perp$ and 7.17 µs for $g_\parallel$. In both cases, the deviation of T_m values between $g_\perp$ and $g_\parallel$ is approx. 2.6%, which is by far smaller than the differences induced by the anisotropy of the proton bath. It should be noted that this conclusion is valid specifically for $[Cu_xPd_{1-x}(dbm)_2]$, which shows only a moderate anisotropy of the g tensor and does not generalise to systems with much larger magnetic anisotropies.

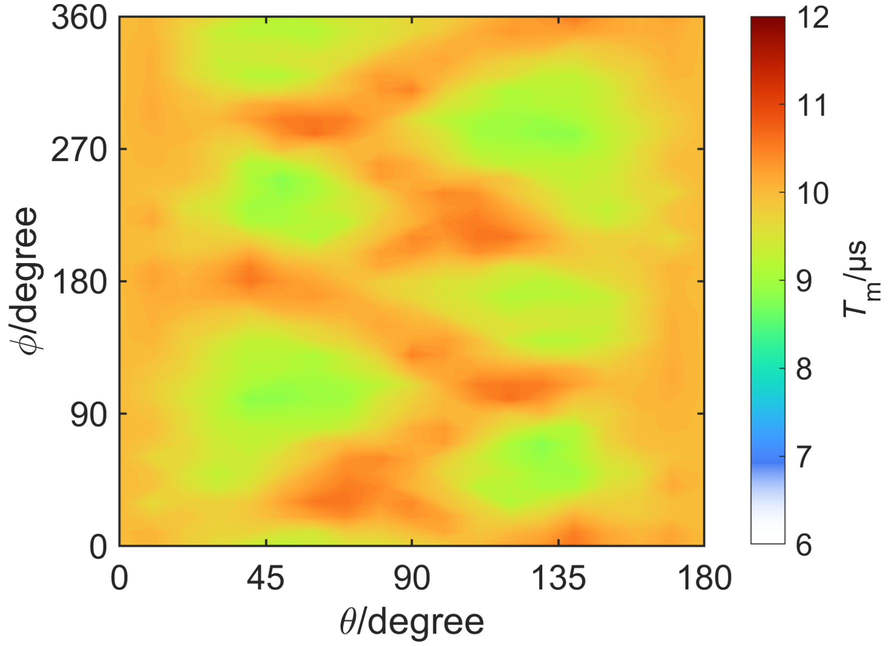

Figure S11: Coherence time T_m of $(PPh_4)_2[Cu_xNi_{1-x}(mnt)_2]$ as a function of the magnetic field orientation. The magnetic field vector was rotated by the polar angle $\theta = 0° \ldots 180°$ and the azimuthal angle $\phi = 0° \ldots 360°$ in 10° steps.

S3.3 Analysis of the nuclear pair contributions of $[Cu_xPd_{1-x}(dbm)_2]$

This section contains the details of the investigation of the crystalline dispersion of 0.001% $[Cu(dbm)_2]$ and its different hypothetical variants with substitution of protons by spin-less nuclei in the isostructural diamagnetic host crystal $Pd(dbm)_2$. The investigated structures are shown in Fig. S12.

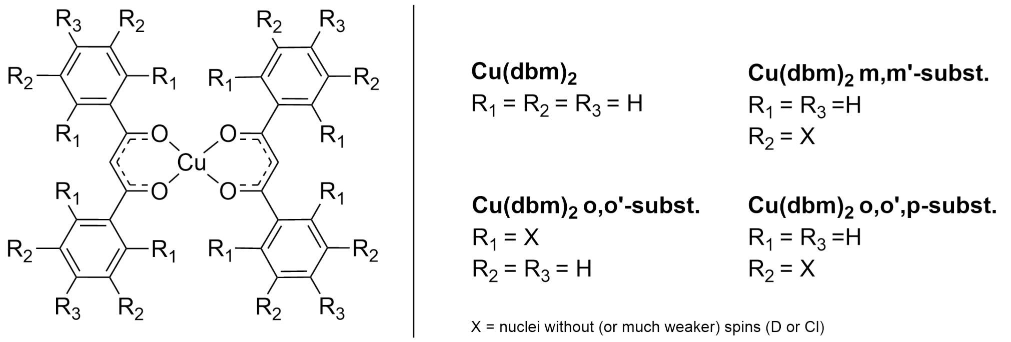

Figure S12: Investigated structures of $[Cu(dbm)_2]$ and and its different hypothetical variants with substitution of protons by spin-less nuclei X.

For these structures, the Hahn echo signal was simulated as described in Sec. S3.1. The Hahn echo decay curves are shown in Fig .S13.

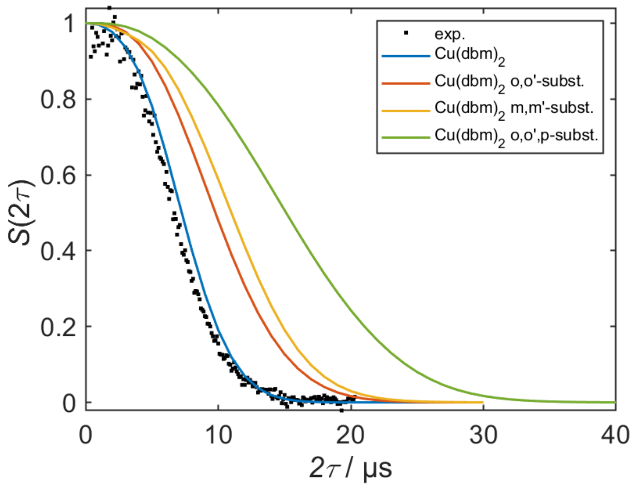

Figure S13: Experimentally measured (symbols, values from Ref. 4) and simulated (lines) coherence decay curves of $[Cu_xPd_{1-x}(dbm)_2]$ (0.001%, $x = 10^{-5}$) and and its different hypothetical variants with substitution of protons by spin-less nuclei.

The Hahn echo decay curve can be fit to the stretched exponential function

$$S(2\tau) = \exp\left\{-\left(\frac{2\tau}{T_\mathrm{m}}\right)^k\right\}, \tag{S22}$$

with the stretch factor k. Based on this stretched exponential function, the coherence time T_m can be determined by fitting the coherence decay curves to this function. In Tab. S1, the stretch factors and the coherence time are summarized for $[\mathrm{Cu}_x\mathrm{Pd}_{1-x}(\mathrm{dbm})_2]$ and its different hypothetical variants with substitution of protons by spin-less nuclei.

Table S1: Stretch factor for the fit of the stretched exponential function for $[\mathrm{Cu}_x\mathrm{Pd}_{1-x}(\mathrm{dbm})_2]$ and different hypothetical variants with substitution of protons by spin-less nuclei.

Molecule	k (sim.)	T_m/µs (sim.)	k (exp.)[a]	T_m/µs (exp.)[a]
$[\mathrm{Cu}_x\mathrm{Pd}_{1-x}(\mathrm{dbm})_2]$	2.7	8.31	2.7	7.74±0.03
o,o'-subst.	2.6	11.33	—	—
m,m'-subst.	2.8	12.54	—	—
o,o',p-subst.	2.6	17.43	—	—

[a] Experimental stretch factor and coherence time from Ref. 4 (fit to stretched exponential).

Fig. S14 and Fig. S15 show the pair contributions analysis and the pair contributions at different times for different hypothetical variants of $[\mathrm{Cu}_x\mathrm{Pd}_{1-x}(\mathrm{dbm})_2]$ with substitution of protons by spin-less nuclei.

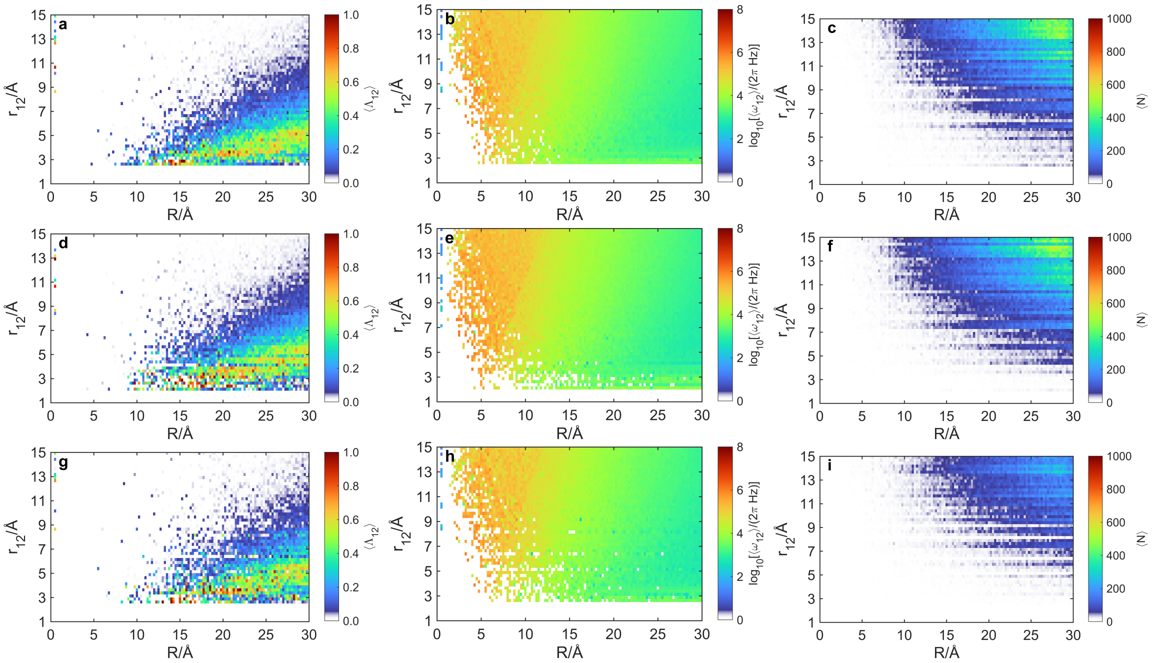

Figure S14: Pair contribution analysis of the spin bath in a substituted [$Cu_xPd_{1-x}(dbm)_2$] crystal. (a)-(c) [$Cu_xPd_{1-x}(dbm)_2$] o,o'-subst., (d)-(f) [$Cu_xPd_{1-x}(dbm)_2$] m,m'-subst., (g)-(i) [$Cu_xPd_{1-x}(dbm)_2$] o,o',p-subst. First column: Modulation depth, second column: modulation frequency, third column: number of protons. Graphs of the first and second column show averages over the respective 0.25 Å × 0.25 Å bins, third column shows the accumulated number of protons within each bin.

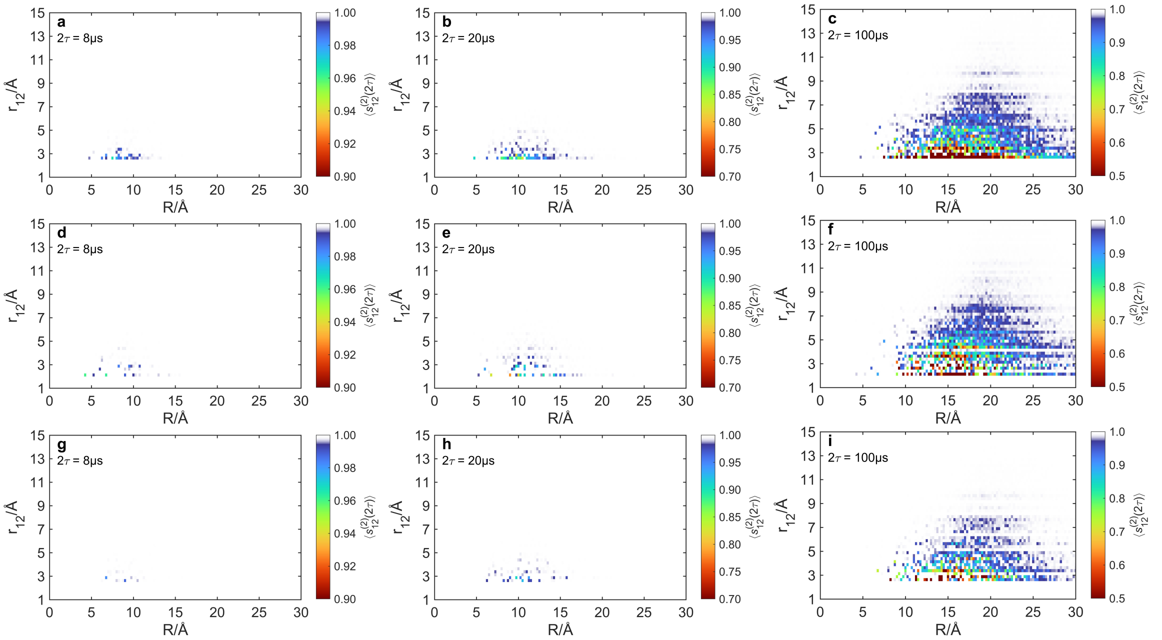

Figure S15: Pair contributions to the Hahn-echo decay. (a)-(c) $[Cu_xPd_{1-x}(dbm)_2]$ o,o'-subst., (d)-(f) $[Cu_xPd_{1-x}(dbm)_2]$ m,m'-subst., (g)-(i) $[Cu_xPd_{1-x}(dbm)_2]$ o,o',p-subst. for delay times as indicated in the panels. Note that the colour scale has been adapted for the various time steps to enhance visibility.

S3.4 Analysis of the nuclear pair contributions of $(PPh_4)_2[Cu_xNi_{1-x}(mnt)_2]$

This section contains the details of the investigation of the crystalline dispersion of 0.001% $(PPh_4)_2[Cu(mnt)_2]$ and its different hypothetical variants with substitution of protons by spin-less nuclei in the isostructural diamagnetic host crystal $(PPh_4)_2[Ni(mnt)_2]$. The investigated structures are shown in Fig. S16.

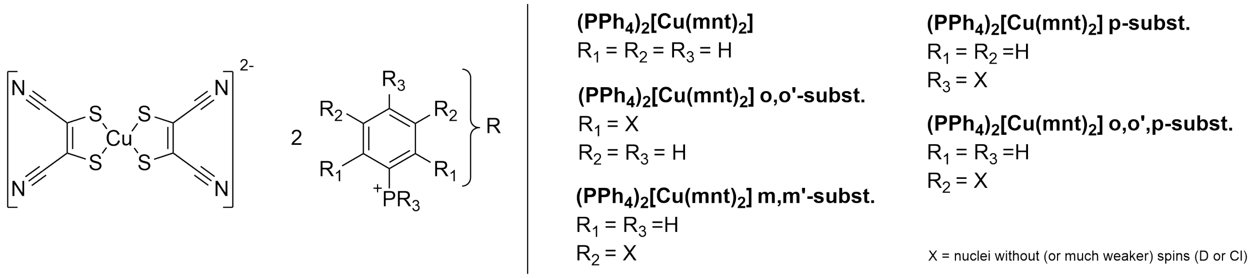

Figure S16: Investigated structures of $(PPh_4)_2[Cu(mnt)_2]$ and and its different hypothetical variants with substitution of protons by spin-less nuclei X.

Analogous to $[Cu_xPd_{1-x}(dbm)_2]$, the Hahn echo signal was also simulated for these structures. The Hahn echo decay curves are shown in Fig .S17.

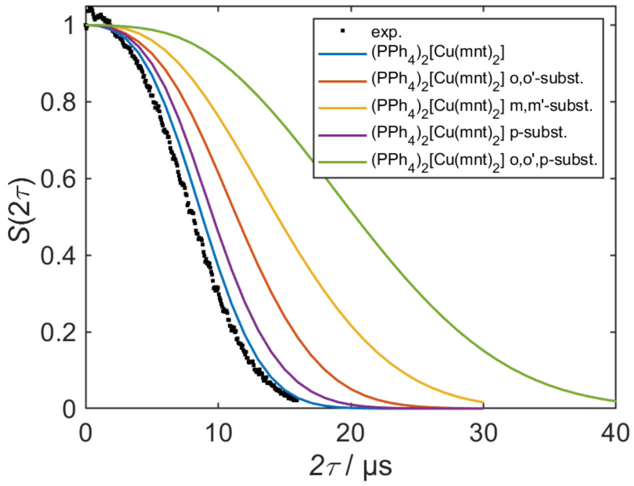

Figure S17: Experimentally measured (symbols, values from Ref. 5) and simulated (lines) coherence decay curves of $(PPh_4)_2[Cu_xNi_{1-x}(mnt)_2]$ (0.001%, $x = 10^{-5}$) and and its different hypothetical variants with substitution of protons by spin-less nuclei.

Using eq. S22, the coherence time of these systems was determined. The stretch factor

and the coherence time are summarized in Tab. S2.

Table S2: Stretch factor for the fit of the stretched exponential function for $(PPh_4)_2[Cu_xNi_{1-x}(mnt)_2]$ and different hypothetical variants with substitution of protons by spin-less nuclei.

Molecule	k (sim.)	T_m/µs (sim.)	k (exp.)[a]	T_m/µs (exp.)[a]
$(PPh_4)_2[Cu_xNi_{1-x}(mnt)_2]$	2.8	10.05	2.5	9.23±0.01
o,o'-subst.	2.7	13.12	—	—
m,m'-subst.	2.5	16.85	—	—
p-subst.	2.8	11.03	—	—
o,o',p-subst.	2.7	23.48	—	—

[a] Experimental stretch factor and coherence time from Ref. 5 (fit to stretched exponential).

Fig. S18 and Fig. S19 show the pair contributions analysis and the pair contributions at different times for $(PPh_4)_2[Cu_xNi_{1-x}(mnt)_2]$ and different hypothetical variants with substitution of protons by spin-less nuclei.

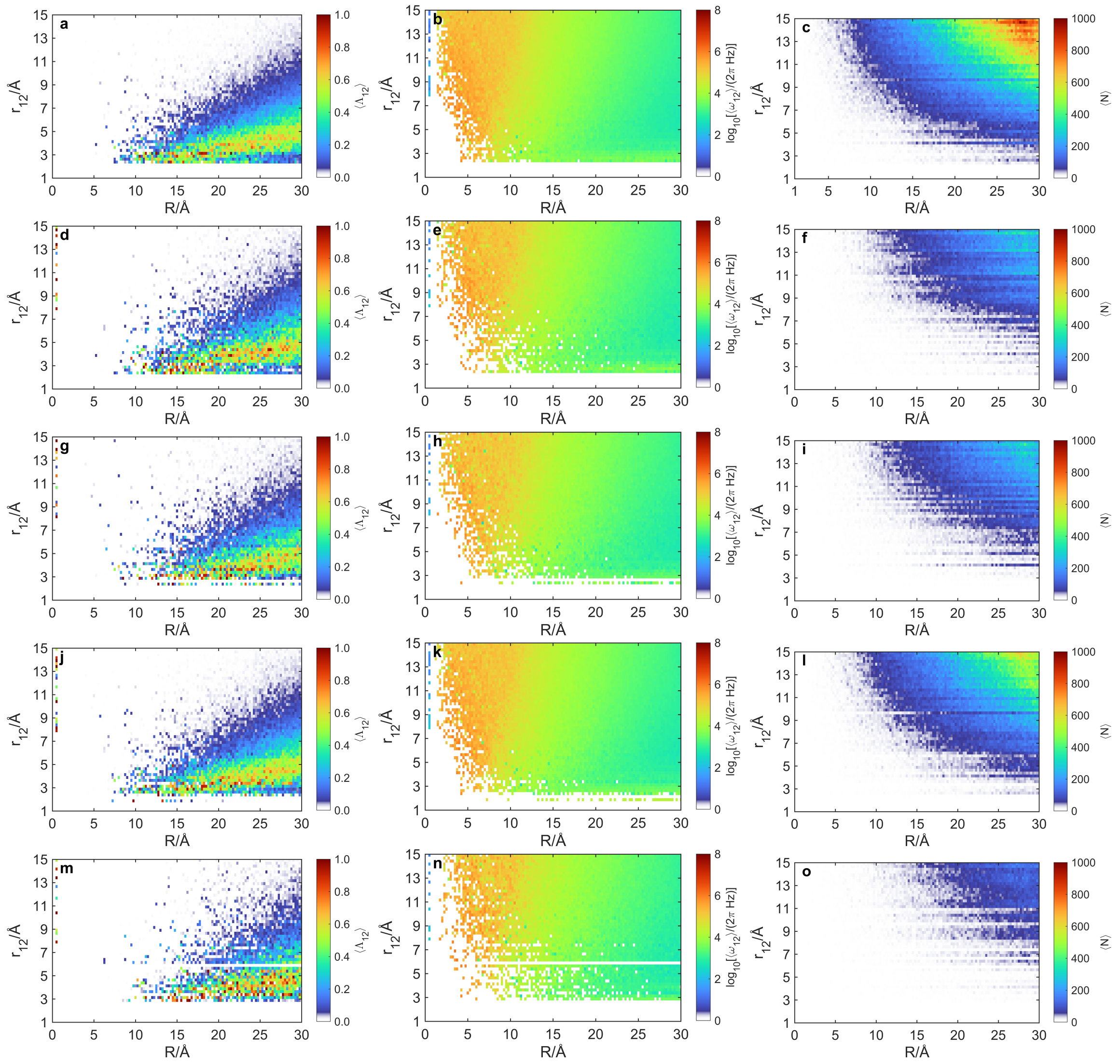

Figure S18: Pair contribution analysis of the spin bath in a $(PPh_4)_2[Cu_xNi_{1-x}(mnt)_2]$ crystal and its different hypothetic variants with substitution of protons by spin-less nuclei. (a)-(c) $(PPh_4)_2[Cu_xNi_{1-x}(mnt)_2]$, (d)-(f) $(PPh_4)_2[Cu_xNi_{1-x}(mnt)_2]$ o,o'-subst., (g)-(i) $(PPh_4)_2[Cu_xNi_{1-x}(mnt)_2]$ m,m'-subst., (j)-(l) $(PPh_4)_2[Cu_xNi_{1-x}(mnt)_2]$ p-subst., (m)-(o) $(PPh_4)_2[Cu_xNi_{1-x}(mnt)_2]$ o,o',p-subst. First column: Modulation depth, second column: modulation frequency, third column: number of protons. Graphs of the first and second column show averages over the respective 0.25 Å × 0.25 Å bins, third column shows the accumulated number of protons within each bin.

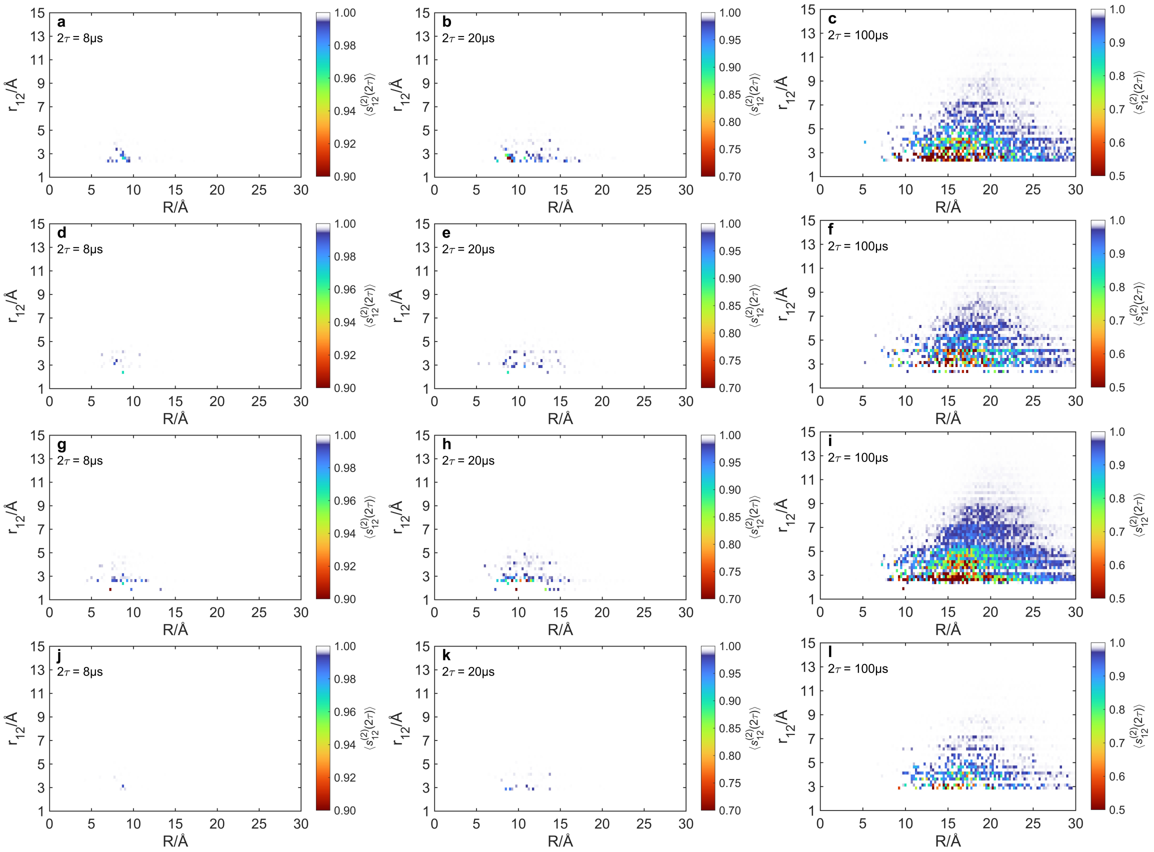

Figure S19: Pair contributions to the Hahn-echo decay. (a)-(c) $(PPh_4)_2[Cu_xNi_{1-x}(mnt)_2]$ o,o'-subst., (d)-(f) $(PPh_4)_2[Cu_xNi_{1-x}(mnt)_2]$ m,m'-subst., (g)-(i) $(PPh_4)_2[Cu_xNi_{1-x}(mnt)_2]$ p-subst. (j)-(l) $(PPh_4)_2[Cu_xNi_{1-x}(mnt)_2]$ o,o',p-subst. for delay times as indicated in the panels. Note that the colour scale has been adapted for the various time steps to enhance visibility.

S3.5 Test of the high-field approximation

The Hahn echo decay of a single electron spin with a bath of spin-$\frac{1}{2}$ nuclei can also be computed by a cluster-correlation expansion (CCE) with a full numerical solution of the time-dependent Schrödinger equation for the subsystems. We followed the generalised CCE approach of Onizhuk and Galli,[6] employing our own implementation in Julia. As an example case, we used $[\mathrm{Cu}_x\mathrm{Pd}_{1-x}(\mathrm{dbm})_2]$ with the magnetic field oriented in the xy-plane (relative to the magnetic main axis system), i.e. $\theta = 90°$ and $\phi = 30°$, see also Sec. S3.2.

The results presented in Fig. S20 show that the APPA accounts very well for the pair contributions at any field strength from 0.1 T to 10 T. The main difference comes from the gCCE1 contributions, which describe the contributions of single nuclear spins due to the anisotropy of the hyperfine coupling. As the simulations clearly show, the gCCE1 signal decays quickly with increasing magnetic field, while the decay of the signal is solely described by the gCCE2 contribution. Interestingly, even at low fields, the gCCE2 contribution is very well reproduced by its high-field approximation (APPA).

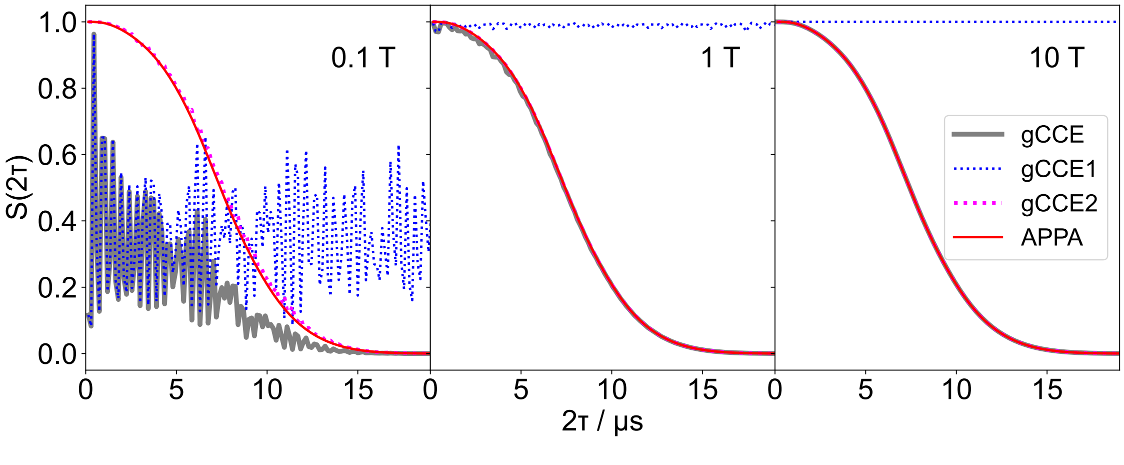

Figure S20: Comparison of the APPA versus a full numerical solution of the gCCE (up to order 2). The latter allows to also model the impact of the magnetic field strength (indicated in each panel). gCCE is the total simulated signal and gCCE1 and gCCE2 are the individual single-nucleus and pair contributions.

References

(1) Witzel, W. M.; Sousa, R. d.; Sarma, S. D. Quantum theory of spectral-diffusion-induced electron spin decoherence. *Phys. Rev. B* **2005**, *72*, 161306.

(2) Witzel, W. M.; Sarma, S. D. Quantum theory for electron spin decoherence induced by nuclear spin dynamics in semiconductor quantum computer architectures: Spectral diffusion of localized electron spins in the nuclear solid-state environment. *Phys. Rev. B* **2006**, *74*, 035322.

(3) (a) Jeschke, G. Nuclear pair electron spin echo envelope modulation. *J. Magn. Reson. Open* **2023**, *14*, 100094; (b) Jeschke, G. Erratum: Nuclear pair electron spin echo envelope modulation. *J. Magn. Reson. Open* **2023**, *14*, 100115.

(4) Lenz, S.; Bader, K.; Bamberger, H.; van Slageren, J. Quantitative prediction of nuclear-spin-diffusion-limited coherence times of molecular quantum bits based on copper(ii). *Chem. Commun.* **2017**, *53*, 4477 – 4480.

(5) Bader, K.; Dengler, D.; Lenz, S.; Endeward, B.; Jiang, S.-D.; Neugebauer, P.; van Slageren, J. Room temperature quantum coherence in a potential molecular qubit. *Nat. Commun.* **2014**, *5304*, 2041–1723.

(6) Onizhuk, M.; Galli, G. PyCCE: A Python Package for Cluster Correlation Expansion Simulations of Spin Qubit Dynamics. *Adv. Theory Simul.* **2021**, *4*, 2100254.
